# A systematic review of economic evaluation of healthcare associated infection prevention and control interventions in long term care facilities

**DOI:** 10.1186/s13561-024-00582-8

**Published:** 2024-11-29

**Authors:** Eric Nguemeleu Tchouaket, Fatima El-Mousawi, Stephanie Robins, Katya Kruglova, Catherine Séguin, Kelley Kilpatrick, Maripier Jubinville, Suzanne Leroux, Idrissa Beogo, Drissa Sia

**Affiliations:** 1 Canadian Research Chair in the Economics of Infection Prevention and Control, Department of Nursing, Université du Québec en Outaouais, St-Jérôme Campus 5, rue Saint-Joseph, Office, Saint-Jérôme, Québec J-2204, J7Z 0B7 Canada; 2 Susan E. French Chair in Nursing Research and Innovative Practice, Ingram School of Nursing, McGill University, 680 Sherbrooke St West, Montréal, Québec H3A 2M7 Canada; 3 School of Nursing, University of Ottawa, 451 Smith Road, Ottawa, ON K1H 8M5 Canada

## Abstract

**Background:**

Healthcare-associated infections (HCAI) are common in long-term care facilities (LTCF) and cause significant burden. Infection prevention and control (IPC) measures include the clinical best practices (CBP) of hand hygiene, hygiene and sanitation, screening, and basic and additional precautions. Few studies demonstrate their cost-effectiveness in LTCF, and those that do, largely focus on one CBP. An overarching synthesis of IPC economic analyses in this context is warranted. The aim of this paper is to conduct a systematic review of economic evaluations of CBP applied in LTCF.

**Methods:**

We twice queried CINAHL, Cochrane, EconLit, Embase, Medline, Web of Science and Scopus for studies published in the last three decades of economic evaluations of CBP in LTCF. We included controlled and randomized clinical trials, cohort, longitudinal, follow-up, prospective, retrospective, cross-sectional, and simulations studies, as well as those based on mathematical or statistical modelling. Two reviewers conducted study selection, data extraction, and quality assessment of studies. We applied discounting rates of 3%, 5% and 8%, and presented all costs in 2022 Canadian dollars. The protocol of this review was registered with Research Registry (reviewregistry1210) and published in BMC Systematic Reviews.

**Findings:**

We found 3,331 records and then 822 records; ten studies were retained. The economic analyses described were cost-minimization (*n* = 1), cost-benefit (*n* = 1), cost-savings (*n* = 2), cost-utility (*n* = 2) and cost-effectiveness which included cost-utility and cost-benefit analyses (*n* = 4). Four studies were high quality, three were moderate, and three were low quality. Inter-rater agreement for quality assessment was 91⋅7%. All studies (*n* = 10) demonstrated that CBP associated with IPC are clinically effective in LTCF and many (*n* = 6) demonstrated their cost effectiveness.

**Interpretation:**

Ongoing economic evaluation research of IPC remains essential to underpin healthcare policy choices guided by empirical evidence for LTCF residents and staff.

**Supplementary Information:**

The online version contains supplementary material available at 10.1186/s13561-024-00582-8.

## Introduction

Healthcare-associated infections (HCAI) are defined as “a localized or systemic condition resulting from an adverse reaction to the presence of an infectious agent(s) or its toxin(s)” occurring within a health care setting [1]. HCAI present a public health concern as they financially burden care institutions, governments, and society and importantly, they reduce quality of life [2–6]. In Europe, 8⋅9 million distinct HCAI episodes are estimated to occur annually in acute and long-term care [7]. As these infections are largely preventable, their incidence is seen as a reflection of the quality of care received [8]. 

Residents of long-term care facilities (LTCF) are at greater risk of contracting HCAI as they are inherently vulnerable due to: comorbidities, decreased immunity, functional impairment, or indwelling devices; sharing common spaces that promotes pathogen transmission and; therapy-related processes such as the widespread use of broad-spectrum antibiotics [1, 2]. Infection prevention and control (IPC) programs outline established practices to protect residents, healthcare staff and the surrounding community from infection [9, 10]. These include transverse clinical best care practices (CBP) that apply to all care settings: (1) hand hygiene; (2) hygiene and sanitation of surfaces and equipment; (3) screening on admission of residents who are carriers or at risk, and; (4) basic and additional precautions such as isolation and personal protective equipment [11]. 

IPC measures have associated costs; therefore, it is important to assess the expected health benefits and financial implications of prevention strategies [12]. In acute care settings, economic evaluations have shown IPC measures can be clinically and cost-effective [13, 14]. In long-term care settings, a scant body of literature exists on economic analyses of interventions aimed at limiting the spread of HCAIs. One 2016 systematic review describes nine studies of IPC in the elderly, however, the only CBP evaluated was hand hygiene [15]. Another review assessed the cost benefits of a screening strategy for tuberculosis (TB) [16]. 

This study will synthesize the existing scientific literature describing economic evaluations of IPC measures using CBP in LTCF. The aim of this study is to conduct a systematic review of economic evaluations of IPC in LTCF that analyzed any of the four CBPs described above.

## Methods

### Protocol registration

A detailed research protocol for this systematic review was previously published [17]. The protocol of this study was registered in Research Registry (reviewregistry1210) and complies with the Preferred Reporting Items for Systematic Reviews and Meta-analyses [18] (PRISMA) statement.

We synthesized the following economic analyses: cost-minimization (CMA), cost-effectiveness (CEA), cost-utility (CUA), cost-benefit (CBA) and cost-consequence (CCA) [17, 19]. Costs were discounted to bring all monetary values to 2022 Canadian dollars (CAD). The published protocol of this study details the theoretical approach and data analyses [17]. 

### Eligibility criteria

The inclusion and exclusion criteria were based on the Population, Interventions, Comparators and designs, Outcomes and Time (PICOT) framework, summarized in Table 1.

**Table 1:** Inclusion and exclusion criteria based on Population, intervention, comparators and designs, outcomes and time (PICOT) framework

	Included	Excluded
**Population**		
Geographic area	All countries	
Establishment	Long-term care: nursing homes, assisted-living facilities, homes for the aged, retirement homes	
Residents	All residents of LTCFs	
Infections	Influenza viruses, noroviruses, *Salmonella* sp., Group A *Streptococcus*, *Sarcoptes scabei*, *Clostridium difficile*, *Escherichia coli*, *Streptococcus pneumoniae*, Respiratory syncytial virus (RSV), *Legionella* spp., Parainfluenza viruses, *Mycobacterium tuberculosis*, Adenoviruses (epidemic keratoconjunctivitis), Hepatitis B virus, *Clostridium perfringens*, Rhinoviruses, *Chlamydia pneumoniae*, *Shigella* sp., Methicillin-resistant *Staphylococcus aureus* (MRSA), Coronaviruses (SARS-CoV-2), Rotaviruses, *Campylobacter* sp., Trichophyton	
**Interventions** Clinical best practices (CBPs)	Hand hygiene; hygiene and sanitation; screening on admission; basic and additional precautions	Antibiotics, any other medications
**Comparators and designs**	Quantitative studies: controlled clinical trials, randomised clinical trials, cohort studies, longitudinal studies, follow-up studies, prospective studies, retrospective studies, cross-sectional studies, mathematical/statistical modelling, and simulations	Qualitative studies, literature reviews (systematic reviews, meta-analyses, meta-syntheses, scoping reviews)
**Outcomes** Types of economic evaluation Economic evaluation’s measures	Cost-minimization analysis, cost-effectiveness analysis, cost-utility analysis, cost-benefit analysis, or cost-consequences analysis Costs estimates of CBPs, incremental cost-effectiveness ratio, incremental cost per quality-adjusted life-year, incremental cost per disability-adjusted life-year and the incremental cost-benefit ratio, net costs, and net cost savings	Technological assessments, purely clinical studies, pharmacological studies

### Population (P)

This review included studies in long-term care settings in any country.

### Interventions (I)

Studies were restricted to four CBPs in IPC programs: (1) hand hygiene; (2) hygiene and sanitation of surfaces and equipment; (3) screening of residents according to established protocols; and (4) basic and additional precautions [20]. Pharmacoeconomic studies, technological assessments, purely clinical studies, and pharmacological studies were excluded.

### Comparators and designs (C)

We included the following quantitative studies: controlled clinical trials, randomized clinical trials, cohort, longitudinal, follow-up, prospective, retrospective, cross-sectional, simulations, and studies based on mathematical or statistical modelling. Qualitative studies were excluded. No comparator was specified.

### Outcomes (O)

Outcomes included all quantitative studies using CMA, CEA, CUA, CBA), or CCA, and those combining any of these types of analyses.

### Time frame (T)

Articles were included if they were published in the last three decades, between January 1990 and September 1st, 2023, inclusively.

### Data sources and research strategy

The research strategy is presented here and has been previously published [17]. Keywords for the search strategy were chosen by two IPC program specialists, co-authors (ET, FEM, SR, KKR) and a librarian at the Saint-Jérôme Campus of the Université du Québec en Outaouais (CS). We queried: CINAHL, Cochrane, EconLit, Embase, Medline, Web of Science and Scopus (see Supplementary material 1–7 for search strategies).

### Selection process

The search was performed on March 7th, 2022, and updated on September 1st, 2023. All records were imported to Endnote, duplicates were removed, and the database was then imported into Rayyan [21], a review screening platform. To pilot test the screening strategy and ensure reliability, an identical sample of 10% of the records was first assessed by two co-authors (FEM, SR). Three co-authors (ET, FEM, SR) then met to resolve any conflicts and to standardize the interpretation of the screening strategy. One reviewer (FEM) screened the titles and abstracts of all records. The records were divided between three reviewers (ET, KKR, SR) for screening. Papers were included if at least two independent reviewers agreed it met inclusion criteria. In case of disagreements, a third reviewer’s decision broke the conflict.

### Data extraction

Data from included studies were extracted to an Excel spreadsheet based on the Consolidated Health Economic Evaluation Reporting Standards (CHEERS) [22]. The spreadsheets were developed by three co-authors (ET, FEM, SR), and reported: authors names, year of publication, country, CBPs studied, infections targeted, study design, population, setting, period of data collection and whether it was an outbreak period. Further information included: economic evaluation method, analysis perspective, time horizon, currency, and whether discounting and sensitivity analysis were performed. When time horizon was not mentioned we considered the data collection period as the time horizon. Data were extracted for costs and outcomes of intervention and control groups. All incremental costs, outcomes, cost-savings, effectiveness-cost and benefit-cost ratios and their interpretation were extracted. When analyses were not reported, calculations were performed using the available data.

### Quality assessment

Our team used three quality assessment tools commonly used in economic evaluation assessments. The first tool used was the checklist for economic evaluations by the Scottish Intercollegiate Guidelines Network (SIGN) [23], the second was the Drummond critical assessment for economic evaluations [24], and the third was the Cochrane Handbook for Systematic Reviews of Interventions [25]. The assessments were conducted by one co-author (FEM) and the score was expressed as a percentage. Another co-author (SR) assessed the quality for two studies, and the inter-rater agreement (IRA) percentage was calculated. Raters’ agreement was given a value of 1, disagreement a value of 0. The values for all the statements were summed and then divided by the total number of statements for each quality assessment tool to obtain the IRA percentage. To standardize the evaluation and avoid interpretation biases, a third co-author (ET) was consulted to agree on the interpretation of each statement of the quality assessment tools. The evaluations were then adjusted based on this standardized interpretation. For every study, the quality assessments of the three tools were averaged and reported as a percentage of positive (yes) or high answers, moderate answers, or negative (no) or low answers. As done in our previous publication [14], the overall quality was then determined to be “High quality” if the average quality assessment score of high (yes) answers was over 80%, “Moderate quality” if the score was between 60% and 79⋅9%, and “Low quality” if the average score was below 60%.

### Data analysis

The Dominance Ranking Matrix classification tool [26] was used to interpret the results included in the review to determine whether interventions should be rejected, favored, or it remained unclear whether it should be rejected or favored. For discounting, we first converted all monetary data to 2022 CAD using the relevant benchmark exchange rates of the Bank of Canada. A discounting rate of 3%, 5% and 8% was then applied. Effects were considered to remain stable over time and were therefore not discounted.

### Role of the funding source

The funder of the study had no role in study design, data collection, data analysis, data interpretation, or writing of the report.

## Results

**Fig. 1:**
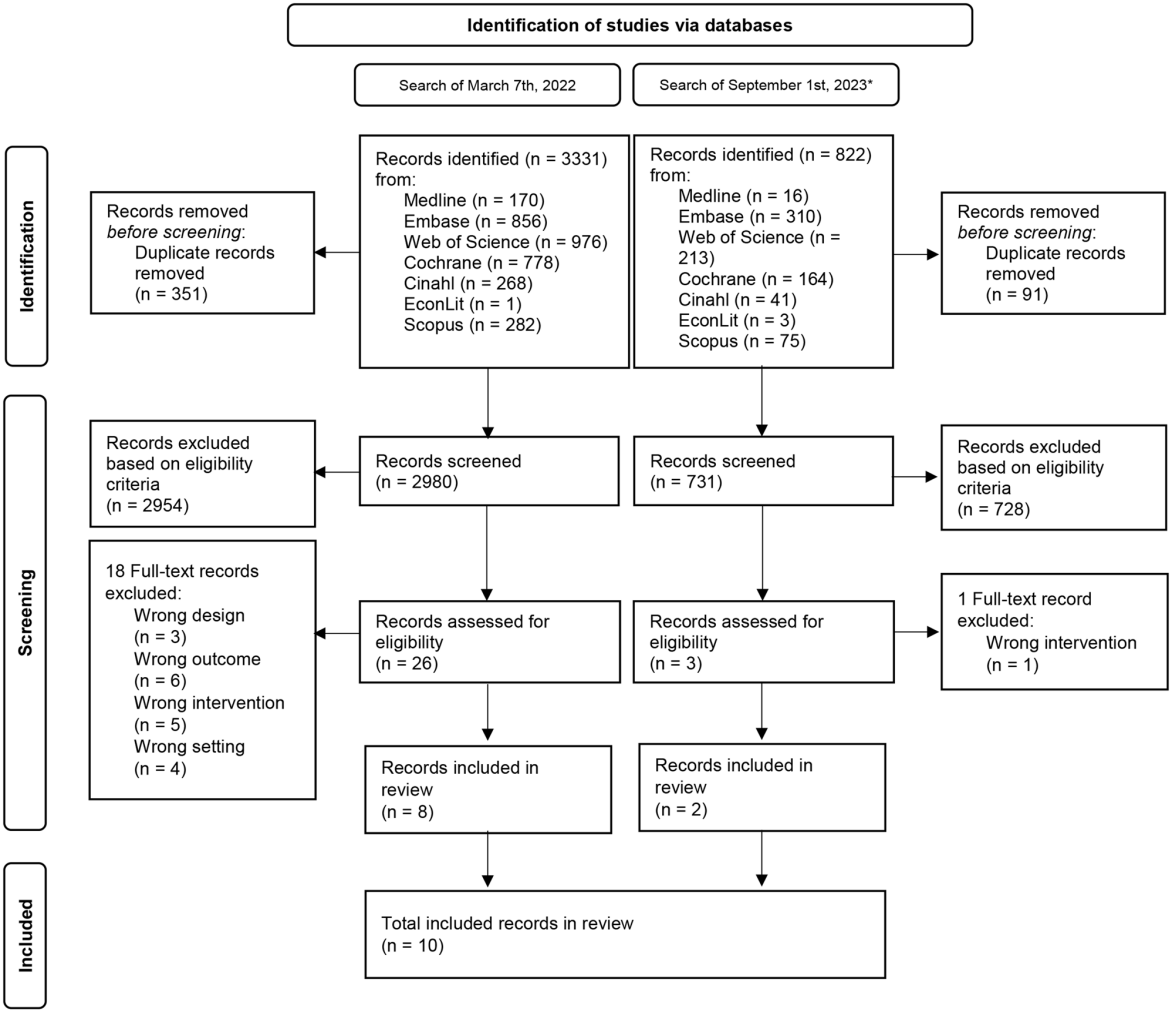
Flow diagram of study selection to include in the systematic review. *Search limited to records published in 2022 and 2023

The first search of all databases yielded 3,331 records, of which 351 were duplicates and removed, leaving 2,980 records to screen. Following screening, 26 records remained, of which 20 required conflict resolution. After excluding 18 records, eight studies were included in the review (Fig. 1). The update search yielded 822 records, of which 91 were duplicates and removed, leaving 731 records. Following screening, 13 records remained, of which ten required conflict resolution. After excluding 11 records, two studies were included in the review (Fig. 1). A list of excluded studies after full-text reading is provided in Supplement 8.

Table 2 provides a summary of the included studies based on the CHEERS checklist. The complete CHEERS checklist is available in Supplement 9. One study [27] (10%) was published in 1999, one study [28] (10%) was published in 2002, one study [29] (10%) was published in 2004, one study [30] (10%) was published in 2013, two studies [31, 32] (20%) were published in 2018, one study was published for each of 2020 [33], 2021 [34], 2022 [35] and 2023 [36] (40%). Four studies [27, 28, 30, 33] (40%) were conducted in Canada, four [29, 31, 34, 36] (40%) in the United States of America, one [32] (10%) in Hong Kong and one [35] (10%) in Spain.

**Table 2:** Summary of the Consolidated Health Economic Evaluation Reporting Standards (CHEERS) checklist for the included studies

Authors	Campbell et al. [33]	Church et al. [28]	Hutton et al. [31]	Lee et al. [34]	Li et al. [32]	Marchand et al. [27]	Salmerón et al. [35]	Sansone & Bravo [36]	Trick et al. [29]	Verma et al. [30]
Year of publication	2020	2002	2018	2021	2018	1999	2022	2023	2004	2013
Country	Canada	Canada	United States of America	United States of America	Hong Kong	Canada	Spain	USA	United States of America	Canada
Study perspective	Health system perspective		Health care system perspective	Hospital perspective, Third-Party payer perspective, Societal perspective	Health service provider perspective	Health-care system perspective (Societal perspective because in Canada)				Health care system perspective
Intervention	Active testing of groups at increased risk of acquiring Covid-19 (community health care workers and people at long-term care facilities)	Rapid influenza A virus infection diagnostic service	Targeted infection prevention multimodal intervention program for catheter associated urinary tract infections	Two interventions were modeled: an extensively drug-resistant organism registry plus a CRE prevention bundle	3 screening strategies: TB Xpert screening, TB chest X-ray screening and, LTBI and TB interferon-gamma release assays and chest X-ray screening	Screening with the tuberculin test plus chemoprophylaxis for those at high risk for TB	COVID-19 screening strategy for second wave: serialization of positive serologies for COVID-19 on a quarterly basis in order to avoid performing unnecessary AIDT (PCR or rapid test of antigens), sick leave, and quarantines	Care bundle consisting of 5 components: (1) close monitoring of staff’s hand hygiene compliance when handling residents; (2) routine checking of residents’ hydration status; (3) effective residents’ incontinence and perineal care; (4) in-house UTI treatment and monitor of antibiotic use; and (5) daily updates regarding the bundle implementation progress at morning medical staff huddles	Routine glove-use	Three screening strategies for TB on entry to long term care facilities
Comparators	Status quo (= current strategy) defined based on the testing performed between July 8 and 17, 2020, which includes testing of symptomatic people and limited testing of asymptomatic people (e.g., some individuals with exposure or at high risk of exposure).	6 control nursing homes and 6 experimental nursing homes	6 control nursing homes and 6 intervention nursing homes	No intervention scenario	No screening strategy	Intervention screening strategy and current standard of care (case-finding and treatment approach)		Baseline status	Contact-Isolation Precautions Section	No screening
Study design	Cross-sectional study	Randomized clinical trial	Retrospective analysis of randomized clinical trial	Simulation and modelling	A simplified decision analytic process based on Markov model	Modelling	Retrospective observational study	This Quality assurance and performance improvement initiative consisted of 3 stages: a baseline, an intervention and a follow-up (Crossover design)	Random allocation of two similar sections of the skilled-care unit to one of the infection-control strategies	Modelling
Discount rate	Discounting done, but the rate is not specified.		Discounting done, but the rate is not specified.		The costs and effectiveness outcomes were discounted at an annual rate of 5% and adjusted by half-cycle correction.	5%				3%
Health outcomes	Number of people sampled	For influenza A + residents: illness duration, antibiotic prescription rates (number of times the drug was prescribed, the doses prescribed & duration of the treatment), length of hospital stay Rate of hospitalization (experimental vs. control) Attack rate Mortality rate	QALYs lost related to CAUTI	Number of CRE infections Number of CRE-attributable deaths QALYs lost	LYs and QALYs	Incremental costs per case avoided per life-year and per quality-adjusted life-year; Incremental costs per death avoided per life-year and per quality-adjusted life-year; Annualized measurements of health events and the cost impact of screening an annual number of INH related hepatitis and INH-related deaths, an annual number of TB cases, and TB-related deaths, and the annual cost increment per 1000 institutionalized patients)	Number of PCR tests, sick day leaves and quarantines avoided	UTI rates	Acquisition of microbial organisms (MRSA, extended-spectrum β-lactamase (ESBL)-producing Klebsiella pneumoniae (KP), ESBL-producing Escherichia coli (EC), Vancomycin resistant enterococci) measured by positive cultures	Number of cases
Measurement of effectiveness	Cost-benefit	Cost-effectiveness (cost-utility)	Cost- effectiveness (cost-utility & cost-benefit)	Cost- effectiveness (cost-utility & cost-benefit)	Cost-effectiveness (cost-utility & cost-benefit)	Cost- effectiveness (cost-utility & cost-benefit)	Cost-saving	Cost-saving	Cost-minimization	Cost-effectiveness (cost-utility)
Currency	Canadian dollars	Canadian dollars	United States dollars	United States dollars	United States dollars	Canadian dollars	United States dollars ^a^	United States dollars ^a^	United States dollars ^a^	Canadian dollars
Price date	2020	1999 ^a^	2015	2021 ^a^	2018 ^a^	1992	2020 ^a^	2022 ^a^	1999 ^a^	2010 ^a^
Study findings	Active testing strategies can identify a high proportion of people with SARS-CoV-2 infection and minimal or no symptoms. Our analysis shows that actively testing populations at increased risk of acquiring SARS-CoV-2 in Canada can be feasible. Systematic tracing and testing of 16 contacts per person given a new diagnosis of SARS-CoV-2 infection marginally increases testing costs and could be accomplished with current laboratory capacity.	Our study shows that the new laboratory service provided for the experimental nursing homes significantly diminished the overall duration of outbreaks of influenza A virus infection in nursing homes in the region.	This cost-effectiveness analysis showed that this intervention program was expected to save $34,000 per year and improve health outcomes by 0·2 QALYs.	Targeting these facilities decreased the prevalence of carriage by a relative 17% and 22% regionwide and within Cook County, respectively, regardless of constraints.	Although no screening offered the greatest cost-saving, LTBI/TB screening was the most effective strategy with highest LYs and QALYs gained and more likely to be cost-effective under the willingness to pay threshold of US$50,000 per QALY gained.	Screening improves the health of the average patient in both baseline and sensitivity analysis.	The serological serialization of coronavirus on a quarterly basis, in residents and employees of our nursing home, has proven to be efficient in avoiding unnecessary expenditures during a coronavirus outbreak, as well as avoiding quarantines and sick leave of participants with positive IgG	The implementation of this novel bundle of care was successful in (1) achieving a strong decline in UTIs among residents without indwelling catheters which met the initiative goal; (2) maintaining UTIs lower than the national rate for more than 2 years which validated the efficacy and sustainability of the bundle; (3) avoiding hospitalizations through in-house treatment of residents; and (4) reducing antibiotic use. The sharp decline in UTIs also generated a net saving of $33,907 per quarter by reducing costs of diagnostic and follow-up tests, use of antibiotics, and extra care expenses related to medical equipment and staff	There was a similar frequency of transmission of antimicrobial-resistant bacteria in the two study sections; there was evidence for resident-to-resident KP transmission in the isolation-precautions section. Routine glove use for healthcare workers, which decreases resident social isolation and healthcare facility costs, may be preferable in many long-term care facilities.	Our study found that screening was costly, with large numbers needed to screen to prevent a case. Our analysis indicated that tuberculin skin test screening is more cost-effective than CXR screening for prevalent disease. Screening all entrants to long-term care for TB may not be cost-effective in a low-burden setting.

a: Information inferred by the authors. When the year of currency was not reported, the last year of data collection was considered as the currency year, and when not available, the publication year was considered as the currency year. When currency was not specified, the currency was assumed based on the country of the study

Of the IPC CBP interventions, six studies [27, 28, 30, 32, 33, 35] (60%) described screening strategies, one [29] (10%) examined routine glove use, one [36] (10%) was a care bundle including monitoring of staff’s hand hygiene compliance (10%), and two studies [31, 34] (20%) were of a combination of CBPs (hand hygiene, contact precautions, surveillance, registry tracking, screening, and daily chlorhexidine gluconate bathing). Three studies targeted TB [27, 30, 32] (30%), two targeted COVID-19 [33, 35] (20%), one [29] (10%) targeted four multi-drug resistant bacteria (methicillin resistant *Staphylococcus* aureus, vancomycin-resistant enterococci, extended-spectrum b-lactamase-producing *Klebsiella pneumoniae* and *Escherichia coli*) while the other four studies targeted : catheter associated urinary tract infections (CAUTI) [31] (10%), urinary tract infections [36] (10%), Influenza A [28] (10%), carbapenem-resistant Enterobacteriaceae (CRE) [34](10%).

Four studies [27, 30, 32, 34] (40%) were modelling studies, two [28, 31] were randomized clinical trials (20%), one [33] (10%) was a cross-sectional study, one [35] (10%) was an observational study, one [36] (10%) was a crossover study, and one study [29] (10%) consisted of a random allocation of two similar sections of the facility to one of two different infection control strategies. Sample sizes for non-modelling studies varied between 156 participants [29] and 3,492,250 participants [33]. For one study [28] (10%), data collection was conducted during an outbreak period.

**Fig. 2:**
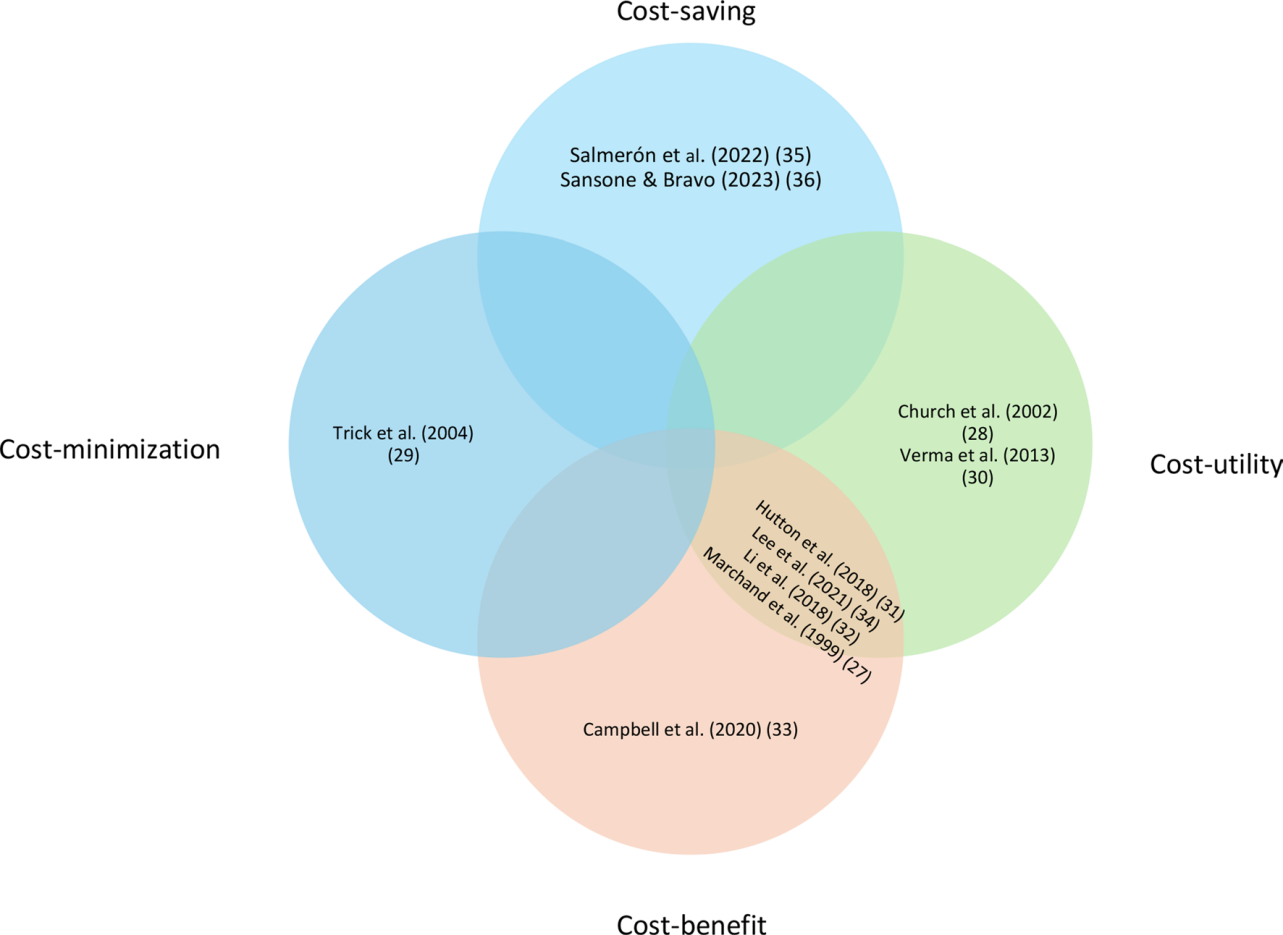
Venn diagram of cost-effectiveness types of the studies included in the systematic review

Four studies [27, 31, 32, 34] (40%) were cost-effectiveness studies combining cost-utility and cost-benefit, two [28, 30] (20%) were cost-utility studies, two [35, 36] (20%) were cost-saving studies, one [33] (10%) was a cost-benefit study, and one [29] (10%) was a cost-minimization study (Fig. 2).

Five studies [27, 30–33] (50%) used a healthcare system perspective. Lee et al. [34] provided results from three perspectives: hospital, third-party payer, and a societal perspective. Time-horizons varied between nine days [33] and 20 years [32]. Three studies [27, 30, 32] (30%) reported discounting of costs along with the corresponding discounting rate. Seven studies [27, 29–34] (70%) performed sensitivity analyses. When the year of currency was not reported, we considered it to be the last year of data collection [28–30, 35, 36], and when not available, the publication year was considered as the currency year [32, 34] (Table 2). When currency was not specified, we assumed the currency based on the country of the study [29, 35, 36] (Table 2).

**Table 3:** Economic evaluation characteristics of all included studies

Authors	Health Measure (outcomes of intervention)		Intervention		Control		Incremental cost (E)	Incremental effect or incremental benefit (F)		Incremental cost-savings (G)	Incremental cost-effectiveness ratio (H)	
			Cost of intervention (A)	Outcomes (effects or benefits) of intervention (B)	Cost of control (C)	Outcomes (effects or benefits) of control (D)	E = A-C	F = B-D	F in $	G = absolute (F in $) – absolute (E)	H = (F/E) * 10^4^ (in health measure/$)	H = (F in $/E) * 10^4^
Campbell et al. (33)	Strategy 3 testing		$124,800,000		$67,800,000		$57,000,000 ^a^		$113,300,000 ^a^	-$56,300,000		$19,877·19 ^a^
	Strategy 3 + Strategy 1 + Status Quo testing		$203,700,000				$135,900,000 ^a^		$218,000,000 ^a^	-$82,100,000		$16,041·21 ^a^
Church et al. (28)	Duration of influenza outbreak (days)		$69·52	9 ^a^	$98·55	16 ^a^	-$29·03	-7 ^a^			2,411·30 ^a^	
Hutton et al. (31)	CAUTI events		$139,948	15·5	$173,986	24·2	-$34,038	-8·7	-$15,136	-$18,902 ^a^	2.56 ^a^	$4,446·79 ^a^
	Hospitalisation due to CAUTI events			6·8		9·7		-2·9	-$39,180	-$5,142 ^a^	0.85 ^a^	$11,510·66 ^a^
	QALYs lost from CAUTI events			0·35		0·55		-0·2				
	Total								54,316	$20,278		-$15,957·45 ^a^
Lee et al. (34)	Number of CRE Infections	All facilities eligible	$303,300,000	2,896	$325,500,000	3186	-$22,200,000 ^a^	-290 ^a^			0·13 ^a^	
		Geographic constraint	$298,400,000	2,853			-$27,100,000 ^a^	-333 ^a^			0·12 ^a^	
	Number of CRE-Attributable Deaths	All facilities eligible	$303,300,000	348		383	-$22,200,000 ^a^	-35 ^a^			0·02 ^a^	
		Geographic constraint	$298,400,000	343			-$27,100,000 ^a^	-40 ^a^			0·01 ^a^	
	QALYs Lost	All facilities eligible	$303,300,000	3,679		4,049	-$22,200,000 ^a^	-370 ^a^			0·17 ^a^	
		Geographic constraint	$298,400,000	3,626			-$27,100,000 ^a^	-423 ^a^			0·16 ^a^	
Li et al. (32)	LYs gained	Xpert	$162	11·1952	$121	11·1907	$41	0·0045			1·1 ^a^	
		CXR	$168	11·1942			$47	0·0035			0·74 ^a^	
		LTBI/TB screening	$430	11·2003			$309	0·0096			0·31 ^a^	
	QALYs gained	Xpert	$162	11·1702		11·1634	$41	0·0068			1·66 ^a^	
		CXR	$168	11·1687			$47	0·0053			1·13 ^a^	
		LTBI/TB screening	$430	11·1792			$309	0·0158			0·51 ^a^	
Marchand et al. (27)	Cases of TB predicted (conversion rate 0.6%)		$6,100	0·52	$3,204	1·28	$2,896	-0·76			-2·62 ^a^	
	TB-related deaths predicted (conversion rate 0.6%)			0·24		0·55		-0·31			-1·07 ^a^	
	Cases of TB predicted (conversion rate 0)		$5,965	0·52	$2,283	0·95	$3,682	-0·43			-1·17 ^a^	
	TB-related deaths predicted (conversion rate 0)			0·21		0·40		-0·19			-0·52 ^a^	
Salmerón et al. (35)	PCR and quarantines avoided									$14,753·5		
Sansone and Bravo (36)	UTI rate per quarter		$23,125	2·3%	$33,907	3·3% ^a^	-$10,782 ^a^	-1% ^a^			0·01 ^a^	
Trick et al. (29)	MRSA case patients’ percentage among those at risk		$2,415	20%	$6,066	18%	-$3,651 ^a^	2% ^a^			-0·05 ^a^	
	ESBL KP case patients’ percentage among those at risk			10%		17%		-7% ^a^			0·19 ^a^	
	ESBL EC case patients’ percentage among those at risk			15%		11%		4% ^a^			0·11 ^a^	
	VRE case patients’ percentage among those at risk			6·8%		8·1%		-1·3% ^a^			0·04 ^a^	
Verma et al. (30)	Cases/1000 entrants - LTBI screening		$202,822	4·6	$124,416	5·3	$78,405	-0·7 ^a^			-0·000009 ^a^	
	Cases/1000 entrants - Active TB screening		$655,649	4·5		5·3	$531,233	-0·8 ^a^			-0·000002 ^a^	

a: Data calculated by the authors based on the data extracted from the studies

Table 3 summarizes the economic evaluation characteristics of all included studies. The four studies [27, 30, 32, 34] (40%) that calculated an incremental cost-effectiveness ratio (ICER) were the modelling studies. One study [31] (10%) reported calculating an ICER, although the ratio was not explicitly stated in the paper.

Campbell et al. [33] showed that active screening of SARS-Cov-2 in groups at increased risk of infection using reverse transcription polymerase chain reaction yielded a cost-saving of $56⋅3 million 2020 CAD, and a cost-saving of $82⋅1 million 2020 CAD if implemented with systematic tracing and contact testing. Using discounting rates of 3–8%, active testing of all community healthcare workers, employees and LTCF residents could provide an incremental cost-saving varying between $59 million and $65 million 2022 CAD. If combined with the testing of contacts of people diagnosed with COVID-19, the incremental cost-saving could climb to $87 million and up to $95 million 2022 CAD.

Church et al. [28] found that, compared to standard practice, the use of a rapid Influenza A diagnostic service could save $11,612 1999 CAD annually, with a reduction of 7 days in the overall duration of outbreaks. For each $10,000 2022 CAD saved by preferentially investing in this rapid viral diagnostic service, the outbreak duration was reduced between 2,200 and 2,067 days.

Hutton et al. [31] found that a targeted infection prevention program against CAUTIs would cost $173,986 2015 United States dollars (USD) less than care as usual. The intervention provided a structured interactive educational program for nursing home staff, hand hygiene promotion, pre-emptive barrier precautions when assisting with high-risk activities of daily living, active surveillance for multidrug-resistant organisms and infections, and an infection preventionist supporting monthly data feedback. They conclude that the “intervention is 85% likely to be cost saving and 96% likely to be cost effective at a threshold of $200,000/ [quality-adjusted life-years] (QALYs).” Hence, $34,000 2015 USD per year could be saved by this intervention along with a 0⋅2 increase in QALYs. This intervention could provide a net cost-saving of $31,800 and up to $44,400 2022 CAD in CAUTI events, hospitalisations and QALYs.

Lee et al. [34] demonstrated that a registry for multi drug-resistant organisms and a CRE prevention bundle yielded cost savings of $19⋅3, $7⋅8, and $68⋅7 million 2021 USD from a hospital, third-party payer, and societal perspective respectively. Upon calculation of an ICER, the intervention was found to be cost-effective from all perspectives. For each $10,000 2022 CAD saved through investing in this intervention, there would be up to 0⋅10 less infections, 0⋅01 less deaths and 0⋅12 QALYs gained for discounting rates between 3 and 8%.

Li et al. [32] compared four TB screening strategies (no screening, TB Xpert screening, TB chest X-ray (CXR) screening and, latent tuberculosis infection (LTBI) and TB interferon-gamma release assays and chest X-ray screening). No screening provided the most cost-saving and LTBI/TB screening was the most cost-effective, with an ICER of $32,150 2018 USD per life-year (LY) and $19,712 2018 USD per QALY. Investing $10,000 2022 CAD in this screening strategy could bring a LY increase of 0⋅75 to 0⋅62 for Xpert strategy, 0⋅51 to 0⋅42 for CXR and 0⋅21 to 0⋅18 for LTBI/TB screening for discounting rates of 3–8%. QALYs gained could range from 1⋅14 to 0⋅94 for the Xpert strategy, 0⋅77 to 0⋅64 for CXR and 0⋅35 to 0⋅29 for LTBI/TB screening for discounting rates of 3–8%.

Marchand et al. [27] compared TB screening using the tuberculin test to chemoprophylaxis for high-risk positive reactors to a case-finding and treatment approach. Screening for TB led to an increase in LYs and QALYs. The ICER was, in 1992 CAD, $3,437 and $2,756 respectively per LY and per QALY gained for a conversion rate of 0⋅6%; and $7,552 and $6,158 respectively per LY and per QALY gained for a conversion rate of 0%. Investing $10,000 2022 CAD in this screening could reduce TB between 1⋅08 and 0⋅26 cases for a conversion rate of 0⋅6% or of 1⋅17 for a conversion rate of 0%, both for discounting rates of 3–8%. TB deaths could be reduced between 0⋅44 to 0⋅11 for a conversion rate of 0⋅6% or of 0⋅52 for a conversion rate of 0%, both for discounting rates of 3–8%.

Salmerón et al. [35] evaluated the efficiency of a screening strategy performed quarterly to avoid unnecessary active COVID-19 detection sick leave and quarantine. They found that the screening strategy would save $14,753 2020 USD.

Sansone et al. [36] established a care bundle initiative to target UTIs that included staff hand hygiene monitoring. Compared to the pre-bundle phase, the intervention showed a decrease of $33,907 2022 USD in UTI costs coupled with a 3⋅3% reduction in UTI rates.

Routine glove use by healthcare workers was found to cost less than contact-isolation precautions, with similar rates of infection with antimicrobial-resistant bacteria, according to Trick et al.’s study [29]. Therefore, investing $10,000 2022 CAD in glove use would not reduce case numbers, but it could reduce IPC costs in comparison to contact-isolation measures.

Verma et al. [30] compared three TB screening strategies (no screening, screening for LTBI using the tuberculin skin test, and screening for active disease with a CXR). They found that to prevent one case, 1,410 and 1,266 persons needed to be screened using the tuberculin skin test and CXR, which cost $109,913 and $672,298 2010 CAD respectively. The study found that screening for all new residents is not cost-effective in a low-burden country such as Canada. A $10,000 2022 CAD investment in LTBI screening could reduce TB cases between 0⋅000006 and 0⋅000004 for discounting rates of 3–8%. The reduction would be of 0⋅000001 less cases for active TB screening (CXR) for discounting rates of 3–8%.

**Table 4:** Overview of the quality assessment of studies using SIGN, Drummond and Cochrane criteria

Authors	OVERALL			Quality
	High	Moderate	Low	
Campbell et al. (33)	77·05%	14·66%	8·29%	Moderate quality
Church et al. (28)	53·67%	24·83%	21·50%	Low quality
Hutton et al. (31)	82·01%	13·21%	4·78%	High quality
Lee et al. (34)	65·30%	8·12%	26·59%	Moderate quality
Li et al. (32)	85·04%	5·09%	9·87%	High quality
Marchand et al. (27)	82·81%	11·93%	5·26%	High quality
Salmerón et al. (35)	48·88%	20·22%	30·89%	Low quality
Sansone & Bravo (36)	55·73%	16·41%	27·86%	Low quality
Trick et al. (29)	60·81%	9·87%	29·31%	Moderate quality
Verma et al. (30)	96·49%	1·75%	1·75%	High quality

**Table 5:** Dominance ranking matrix by Joanna Briggs Institute

Dominance Ranking Matrix, JBI	Campbell et al. (33)	Church et al. (28)	Hutton et al. (31)	Lee et al. (34)	Li et al. (32)	Marchand et al. (27)	Trick et al. (29)	Salmerón et al. (35)	Sansone & Bravo (36)	Verma et al. (30)
Delta costs	+	-	-	-	+	+	-	-	-	+
No. of Studies										
Health benefits	+	+	+	+	+	+	0	0	+	+
Implication for decision makers	Unclear	Favor intervention	Favor intervention	Favor intervention	Unclear	Unclear	Favor intervention	Favor intervention	Favor intervention	Unclear

Table 4 provides an overview of the quality assessment of the studies using the SIGN, Drummond, and Cochrane criteria while supplements 10, 11 and 12 provide individual assessments using these same tools. For high quality studies (score of over 80%), there were four studies [27, 30–32] according to SIGN, five [27, 30–33] according to Drummond, and three [30–32] according to Cochrane. Overall, four studies [27, 30–32] (40%) met a minimum of 80% average across the three quality assessment tools and hence were considered of “high quality”. Three studies [29, 33, 34] (30%) had an overall “moderate quality” (average between 60 to 79⋅9%), whereas three studies [28, 35, 36] (30%) had an average score below 60% and were considered of “low quality” (Table 4). IRA percentage for the quality assessment of the two assessed studies was 91⋅7% across the three quality assessment tools.

The Dominance Ranking Matrix results (Table 5) showed that in six studies [28, 29, 31, 34–36] (60%), the applied intervention should be favored over the comparator, whereas for the four other studies [27, 30, 32, 33] (40%), it is unclear whether the intervention should be favored or rejected due to the higher costs of the intervention.

**Table 6:** Incremental cost-savings and incremental effectiveness-cost ratios for every dollar invested in each CBP with respect to its targeted HCAI IPC program

		Discounting rate: 0%		Discounting rate: 3%		Discounting rate: 5%		Discounting rate: 8%	
Study	Health outcome	Incremental cost-savings	Incremental effectiveness-cost ratio (for each $10,000 invested)	Incremental cost-savings	Incremental effectiveness-cost ratio (for each $10,000 invested)	Incremental cost-savings	Incremental effectiveness-cost ratio (for each $10,000 invested)	Incremental cost-savings	Incremental effectiveness-cost ratio (for each $10,000 invested)
Campbell et al. (33)	Number of people tested according to Strategy 3	-$56,300,000		-$59,728,670		-$62,070,750		-$65,668,320	
	Number of people tested according to Strategy 3, Strategy 1 and Status Quo	-$82,100,000		-$87,099,890		-$90,515,250		-$95,761,440	
Church et al. (28)	Duration of influenza outbreak (days)		2,411·30		2,272·88		2,187·12		2,067·30
Hutton et al. (31)	CAUTI events	-$24,170·19	2	-$29,726·29	1·63	-$34,009·89	1·42	-$41,423·46	1·17
	Hospitalisation due to CAUTI	$6,575·13	0·67	$8,086·58	0·54	$9,251·87	0·47	$11,268·62	0·39
	Total	$25,929·70		$31,890·26		$36,485·69		$44,438·95	
Lee et al. (34)	CRE Infections No. (All facilities eligible)		0·10		0·10		0·10		0·10
	CRE Infections No. (Geographic constraint)		0·10		0·10		0·09		0·09
	CRE-Attributable Deaths, No. (All facilities eligible)		0·01		0·01		0·01		0·01
	CRE-Attributable Deaths, No. (Geographic constraint)		0·01		0·01		0·01		0·01
	QALYs Lost (All facilities eligible)		0·13		0·13		0·13		0·12
	QALYs Lost (Geographic constraint)		0·12		0·12		0·12		0·12
Li et al. (32)	LYs gained (Xpert)		0·85		0·75		0·70		0·62
	LYs gained (CXR)		0·57		0·51		0·47		0·42
	LYs gained (LTBI/TB screening)		0·24		0·21		0·20		0·18
	QALYs gained (Xpert)		1·28		1·14		1·05		0·94
	QALYs gained (CXR)		0·87		0·77		0·72		0·64
	QALYs gained (LTBI/TB screening)		0·39		0·35		0·32		0·29
Marchand et al. (27)	Cases of TB (conversion rate 0.6%)		-2·62		-1·08		-0·61		-0·26
	TB-related deaths (conversion rate 0.6%)		-1·07		-0·44		-0·25		-0·11
	Cases of TB (conversion rate 0%)		-1·17		-1·17		-1·17		-1·17
	TB-related deaths (conversion rate 0%)		-0·52		-0·52		-0·52		-0·52
Salmerón et al. (35)	PCR and quarantines avoided		0·03		0·03		0·03		0·03
Sansone & Bravo (36)	UTI rates per quarter	19,791·8		20,997·1		21,820·5		23,085·2	
Trick et al. (29)	MRSA case patients’ percentage among those at risk		-0·04		-0·02		-0·01		-0·01
	ESBL KP case patients’ percentage among those at risk		0·13		0·07		0·04		0·02
	ESBL EC case patients’ percentage among those at risk		-0·07		-0·04		-0·02		-0·01
	VRE case patients’ percentage among those at risk		0·02		0·01		0·01		0·004
Verma et al. (30)	Cases/1000 entrants - LTBI screening		-0·000009		-0·000006		-0·004		-0·000004
	Cases/1000 entrants - Active TB screening		-0·000002		-0·000001		-0·000001		-0·000001

Table 6 presents incremental cost-savings and incremental effectiveness-cost ratios for every dollar invested in each CBP for each included study.

## Discussion

This study conducted a systematic review of economic evaluations of IPC CBP in LTCF. We assessed the interventions’ costs measures and the relative effects on health outcomes. All the included studies agreed on the effectiveness of their IPC intervention in reducing HCAI. Most of the studies demonstrated cost-effectiveness of practicing IPC (Table 5), in agreement with our previous study conducted in acute care [14]. In some cases, it was unclear whether the intervention was cost-effective over the comparator due to being more expensive despite generating greater health benefits (Table 5). It may be useful to compare the results with a willingness to pay threshold (“For example, in Canada, the commonly referenced willingness to pay threshold in healthcare ranges between CAD 20,000 to CAD 100,000 per QALY gained based on Canadian Agency for Drugs and Technologies in Health” [19]). More research is necessary to evaluate the cost-effectiveness of these interventions. One study concluded that screening for TB is not cost-effective in a low-burden country setting [30]. Only modelling and simulation studies calculated an ICER [27, 30, 32, 34]. One study defined an equation to calculate ICER, but the ratio itself was not calculated [31]. Studies should explicitly define the ICER equation, specifying the numerator and denominator to facilitate comparison between studies. We were able to calculate ICER for most of the studies using the information provided in the studies’ results (Table 3). We recommend that future cost-evaluation studies perform the calculation of the ICER as the ICER serves as a standardized metric for comparative analysis, enabling direct comparisons of the cost-effectiveness of various interventions across different populations or health systems. It proves invaluable in informing resource allocation by quantifying the health benefits gained for every dollar invested. By highlighting which interventions offer the greatest health benefits per unit cost, the ICER assists in prioritizing healthcare initiatives. This, in turn, aids policymakers by providing an evidence-based measure of value, guiding decision-making processes effectively. Four of the included studies were modelling studies, therefore, our review reveals the lack of economic analysis studies performed in LTCF. Our study also revealed a lack of CCA studies. Most of the included studies were published between 2018 and 2021 [31–36] (60%), demonstrating the increased recent interest in IPC cost-evaluation studies in LTCF, which has become particularly relevant after the Covid-19 pandemic.

Quality assessment of the studies revealed some limitations. The IRA indicated that it was highly (91⋅7%) likely that raters agreed on the study’s quality. Their evaluation revealed that half of the studies [28, 29, 34–36] did not perform discounting, and 20% [31, 33] did not specify their discounting rates. Discounting is crucial to allow inter-study comparison and should be undertaken in future cost-evaluation studies. Not all the studies explicitly stated their time-horizon, their study perspective, or the year of their currency which are important parameters. In particular, the currency year is fundamental for appropriate discounting calculations and estimations upon which decisions are made. Therefore, our study highlights some weaknesses in previous cost-evaluation studies that should be addressed in future studies.

We also acknowledge that our study has several limitations. The selection protocol included only English and French papers, therefore, publications in other languages could have been missed. Our analyses only included discounting of the costs. Outcomes were considered stable over time and were therefore not discounted. However, outcomes could vary across time. Since our review included different interventions, we could not perform a meta-analysis or a forest plot of the results. It should be kept in mind that the quality assessment tools used are intended for studies performed in a healthcare setting, and not modelling or simulation studies. Therefore, the tools may not have accurately assessed the quality of these studies [27, 30, 32, 34].

Despite these limitations, our review supports the cost-effectiveness of IPC in LTCF to prevent HCAI. All the included studies agreed on the effectiveness of the interventions in terms of improved health outcomes. Due to financial constraints, cost-effectiveness studies are crucial to support the adoption of interventions. Our review highlights the need to perform more research about IPC using a discounting approach.

## Electronic supplementary material

Below is the link to the electronic supplementary material.

Supplementary Material 1

## Data Availability

No datasets were generated or analysed during the current study.

## Abbreviations

CAD: Canadian dollars
CBA: Cost benefit analysis
CBP: Clinical best practices
CCA: Cost consequence analysis
CEA: Cost effectiveness analysis
CHEERS: Consolidated Health Economic Evaluation Reporting Standards
CMA: Cost minimization analysis
CRE: carbapenem-resistant Enterobacteriaceae
CS: Catherine Séguin
CUA: Cost utility analysis
CXR: Chest X-ray
EC: Escherichia coli
ESBL: extended-spectrum β-lactamase
ET: Eric Tchouaket
FEM: Fatima El-Mousawi
HCAI: Healthcare-associated infections
ICER: Incremental cost-effectiveness ratio
IPC: Infection prevention and control
IRA: Inter-rater agreement
KKR: Katya Kruglova
KP: Klebsiella pneumoniae
LTBI: Latent tuberculosis infection
LTCF: Long-term care facilities
LY: Life-years
MRSA: Methicillin-resistant Staphylococcus aureus
PICOT: Population, Interventions, Comparators and designs, Outcomes, Time
PRISMA: Preferred Reporting Items for Systematic Review and Meta-analyses
QALY: Quality-adjusted life-years
SIGN: Scottish Intercollegiate Guidelines Network
SR: Stephanie Robins
TB: Tuberculosis
USD: United States dollars
UTI: Urinary Tract Infections
