# Supplementary material for: A systematic review of economic evaluation of healthcare associated infection prevention and control interventions in long term care facilities
Source: Health Econ Rev. 2024 Nov 29;14:101. doi: 10.1186/s13561-024-00582-8 (PMC11605862; doi:10.1186/s13561-024-00582-8)
Supplement: Supplementary file 1 — Supplementary Material 1 [file 13561_2024_582_MOESM1_ESM.docx]

**Supplementary material**

**Supplement 1**. Medline search strategy

1. (Clostridioides difficile or vancomycin-resistant enterococci).sh.
2. ('clostridium difficile' or 'c difficile' or 'c-difficile' or 'c-diff' or 'c diff' or clostrid* Carbape* or "hospital acquired" or "Cross infection" or nosocomial* or iatrog*).ab. or ('clostridium difficile' or 'c difficile' or 'c-difficile' or 'c-diff' or 'c diff' or clostrid* Carbape* or "hospital acquired" or "Cross infection" or nosocomial* or iatrog*).ti
3. Urinary-Tract Infections.sh. OR Blood-Borne Pathogens.sh.
4. pneumonia.sh.
5. ('Urinary-Tract Infections' or 'Urinary-Tract Infection' or 'Blood-Borne Pathogens' or 'acquired pneumonia' or 'associated pneumonia' or flu or cold).ab,ti.
6. (Gastrointestinalis or Gastrointestinal or gastroenteritis).ab,ti.
7. gastroenteritis.sh.
8. Haemophilus influenzae.sh.
9. Escherichia coli.sh.
10. Salmonella.sh.
11. ('COVID 19' or 'corona virus' or sras-cov-2 or 'Respiratory syncytial virus' or 'Respiratory infection' or 'Respiratory infections').ab,ti.
12. ('Haemophilus influenzae' or 'Respiratory viruses' or 'Influenza viruses' or 'Parainfluenza viruses' or Adenoviruses or 'Escherichia coli' or Shigella or Rotaviruses or Noroviruses or Salmonella or Rhinoviruses or Chlamydia pneumoniae or Enterovirus).ab,ti.
13. (ERV or Enteroc*).ab,ti.
14. (Vancomycin or vancomycin or methicillin resistant staphylococcus aureus).ab,ti.
15. tuberculosis.ab. or tuberculosis.sh. or tuberculosis.ti.
16. methicillin resistant staphylococcus aureus.sh.
17. (Staphylococcus and aureus and methicillin).ab,ti.
18. (Enteroc* AND resistant AND vancomycin).ab,ti.
19. (Bacil* and gram and neg*).ab,ti.
20. (Scabies or "Skin Diseases" or "Skin Diseases Parasitic" or "Lice Infestations" or "Larva Migrans" or Onchocerciasis or "Parasitic Diseases" or "Ectoparasitic Infestations" or "Mite Infestations" or "Connective Tissue Diseases").sh.
21. (Scabies or 'Skin Diseases' or 'Skin Diseases Parasitic' or 'Lice Infestations' or 'Larva Migrans' or Onchocerciasis or 'Parasitic Diseases' or 'Ectoparasitic Infestations' or 'Mite Infestations' or 'Connective Tissue Diseases').ab,ti.
22. ("Conjunctivitis, Acute Hemorrhagic" or "Conjunctivitis, Allergic" or "Conjunctivitis, Viral" or Conjunctivitis or "Conjunctivitis, Bacterial" or "Conjunctivitis, Inclusion").sh.
23. ('Conjunctivitis, Acute Hemorrhagic' or 'Conjunctivitis, Allergic' or 'Conjunctivitis, Viral' or 'Conjunctivitis' or 'Conjunctivitis, Bacterial' or 'Conjunctivitis, Inclusion').ab,ti.
24. ("Legionella pneumophila" or "Legionella" or "Legionella longbeachae").sh.
25. ('Legionella pneumophila' or Legionella or 'Legionella longbeachae).ab,ti.
26. #1 OR #2 OR #3 OR #4 OR #5 OR #6 OR #7 OR #8 OR #9 OR #10 OR #11 OR #12 OR #13 OR #14 OR #15 OR #16 OR #17 OR #18 OR #19 OR #22 OR #23 OR #24 OR #25 OR #26 OR #27
27. (cost* or price or prices or pricing or expenditure or expenditures or expense or expenses or finan* or fees or charges or budget or budgets).ab,ti.
28. ('econom* analysis' or efficienc* or 'cost effect*' or 'cost utility' or 'cost benefi*' or 'cost consequenc*' or 'cost effic*' or 'cost minimizat*').ab,ti.
29. ("cost allocation" or "cost benefit analysis" or "cost control" or "cost savings" or "costs and cost analysis").sh.
30. #29 OR #30 OR #31
31. ("cohort studies" or "longitudinal studies" or "follow up studies" or "prospective studies" or "retrospective studies" or "randomized controlled trial" or "controlled clinical trial" or "case control studies").sh OR ('cohort stud*' or 'longitudinal stud*' or 'follow-up stud*' or 'prospective stud*' or 'retrospective stud*' or 'controlled clinical trial*' or 'Randomized controlled trial*' or RCT or Random* or blind or case*).ab,ti.
32. (Clean* or control or prevention or screen* or wash or protect* or isolation or sanitation or Hand* or Aseptic* or intervent* or Program* or Strateg* or hygiene*).ab,ti. OR ("hand" or "hygiene" or "asepsis").sh.
33. ('Long-Term Care' or 'Assisted-Living Facilities' or 'long-term-care facility' or 'Homes for the Aged' or 'Nursing Homes' or 'nursing home' or 'long-term care' or retirement home).ab,ti.
34. #28 AND #32 AND #33 AND #34 AND #35

**Supplement 2**. Embase search strategy

1. ('clostridium difficile' or 'c difficile' or 'c-difficile' or 'c-diff' or 'c diff' or clostrid* Carbape* or "hospital acquired" or "Cross infection" or nosocomial* or iatrog*).ab,kw,ti.
2. ('Urinary-Tract Infections' or 'Urinary-Tract Infection' or 'Blood-Borne Pathogens' or 'acquired pneumonia' or pneumonia or 'acquired pneumonia' or 'associated pneumonia' or flu or cold).ab,kw,ti.
3. (Gastrointestinalis or Gastrointestinal or gastroenteritis).ab,kw,ti.
4. ('COVID 19' or 'corona virus' or 'Respiratory syncytial virus' or 'Respiratory infection' or 'Respiratory infections).ab,kw,ti.
5. ('Haemophilus influenzae' or 'Respiratory viruses' or 'Influenza viruses' or 'Parainfluenza viruses' or Adenoviruses or 'Escherichia coli' or Shigella or Rotaviruses or Noroviruses or Salmonella or Rhinoviruses or Chlamydia pneumoniae or Enterovirus).ab,kw,ti.
6. urinary tract infections.sh. OR blood borne staphylococcus aureus pneumonia.sh.
7. Urinary-Tract Infections.mp. or exp urinary tract infection/
8. Blood-Borne Pathogens.mp. or exp bloodborne bacterium/
9. Streptococcus pneumonia/ or hospital acquired pneumonia/ or staphylococcal pneumonia/ or health care associated pneumonia/ or pneumonia/ or bacterial pneumonia/ or community acquired pneumonia/ or aspiration pneumonia/ or infectious pneumonia/ or virus pneumonia/ or Escherichia coli pneumonia/
10. viral gastroenteritis/ or exp acute gastroenteritis/ or exp gastroenteritis/ or exp Transmissible gastroenteritis virus/
11. Haemophilus influenzae.mp. or exp Haemophilus influenzae/
12. Escherichia coli.mp. or exp Escherichia coli/
13. exp Salmonella/
14. Cross infection.mp. or exp cross infection/
15. clostridium difficile.mp. or exp Peptoclostridium difficile/
16. vancomycin resistant enterococci.mp. or exp vancomycin resistant Enterococcus/
17. exp antibiotic resistance/ or exp Staphylococcus aureus/ or exp methicillin resistant Staphylococcus aureus/
18. (ERV OR VRE or Enteroc*).ab,kw,ti.
19. (Staphylococcus and aureus and methicillin).ab,kw,ti.
20. (Enteroc* and resistant and vancomycin).ab,kw,ti.
21. (Bacil* and gram and neg*).ab,kw,ti.
22. exp iatrogenic disease/
23. scabies.mp. or exp scabies/ or skin disease.mp. or exp skin disease/ or parasitic skin disease.mp. or exp parasitic skin disease/ or lice infestation.mp. or exp pediculosis/ or larva migrans.mp. or exp larva migrans/ or exp onchocerciasis/ or onchocerciasis.mp. or parasitic diseases.mp. or exp parasitosis/ or Ectoparasitic Infestations.mp. or exp ectoparasitosis/ or Mite Infestations.mp. or exp mite infestation/ or Connective Tissue Diseases.mp. or exp connective tissue disease/
24. exp bacterial conjunctivitis/ or exp acute hemorrhagic conjunctivitis/ or exp allergic conjunctivitis/ or exp viral conjunctivitis/ or conjunctivitis/ or Conjunctivitis.mp. or bacterial conjunctivitis.mp. or acute hemorrhagic conjunctivitis.mp. or allergic conjunctivitis.mp. or viral conjunctivitis.mp.
25. exp tuberculosis/ or tuberculosis.mp.
26. exp Legionella pneumophila/ or exp Legionella/ or exp Legionella longbeachae/ or exp Legionella feeleii/ or Legionella pneumophila.mp. or Legionella.mp. or Legionella longbeachae.mp. or Legionella feeleii.mp.
27. #1 OR #2 OR #3 OR #4 OR #5 OR #6 OR #7 OR #8 OR #9 OR #10 OR #11 OR #12 OR #13 OR #14 OR #15 OR #16 OR #17 OR #18 OR #19 OR #20 OR #21 OR #22 OR #23 OR #24 OR #25 OR #26
28. (cohort studies or longitudinal studies or follow up studies or prospective studies or retrospective studies or randomized controlled trial or controlled clinical trial or case control studies).ab,kw,ti.
29. cohort studies.mp. or exp cohort analysis/
30. longitudinal studies.mp. or exp longitudinal study/
31. prospective studies.mp. or exp prospective study/
32. exp retrospective study/ or retrospective.mp.
33. exp clinical trial/ or exp controlled study/ or exp controlled clinical trial/ or exp randomized controlled trial/
34. #28 OR #29 OR #30 OR #31 OR #32 OR #33
35. (price or prices or pricing or expenditure or expenditures or expense or expenses or finan* or fees or charges or Budget or budgets or cost* or econom* analysis or efficienc* or cost effect* or cost utility or cost benefi* or cost consequenc* or cost effic* or cost minimizat*).ab,kw,ti.
36. economic aspect.mp. or exp economic aspect/
37. #35 OR #36
38. ('Long-Term Care' or 'Assisted-Living Facilities' or 'long-term-care facility' or 'Homes for the Aged' or 'Nursing Homes' or 'nursing home' or 'long-term care' or 'retirement home').ab,kw,ti.
39. nursing home.mp. or exp nursing home/
40. #38 OR #39
41. (hand or aseptic* or intervent* or program* or hygiene* or clean* or control or prevention or screen* or wash or protect* or isolation or sanitation)
42. #27 AND #34 AND #37 AND #40 AND #41

**Supplement 3**. Web of Science search strategy

1. TOPIC: ('clostridium difficile' OR 'c difficile' OR 'c-difficile' OR 'c. difficile' OR 'c diff' OR 'c-diff' OR 'c. diff' OR clostrid* Carbape* OR "hospital acquired" OR "Cross infection" or nosocomial* OR iatrog*)
2. TOPIC:  ('Urinary-Tract Infections' OR 'Urinary-Tract Infection' OR 'Blood-Borne Pathogens' OR 'acquired pneumonia' OR pneumonia OR 'acquired pneumonia' OR 'associated pneumonia' OR flu OR cold OR tuberculosis OR scabies OR conjunctivitis OR legionella)
3. TOPIC: (Gastrointestinalis OR Gastrointestinal OR gastroenteritis)
4. TOPIC: ('Haemophilus influenzae' OR 'Respiratory viruses' OR 'Influenza viruses' OR 'Parainfluenza viruses' OR Adenoviruses OR 'Escherichia coli' OR Shigella OR Rotaviruses OR Noroviruses OR Salmonella OR Rhinoviruses OR Chlamydia pneumoniae OR Enterovirus)
5. TOPIC: ('COVID 19' OR 'corona virus' OR 'Respiratory syncytial virus' OR 'Respiratory infection' OR 'Respiratory infections')
6. TOPIC: (Staphylococcus AND aureus AND methicillin) OR (vancomycin AND resistant AND Enteroc*) OR (Bacil* AND Gram AND Neg*) OR (Carbape*) OR (ERV OR VRE or Enteroc*)
7. #1 OR #2 OR #3 OR #4 OR #5 OR #6
8. TOPIC: (cost* or price or prices or pricing or expenditure or expenditures or expense or expenses or finan* or fees or charges or budget or budgets OR ‘econom* analysis’ or efficienc* or ‘cost effect*’ or ‘cost utility’ or ‘cost benefi*’ or ‘cost consequenc*’ or ‘cost effic*’ or 'cost minimizat*')
9. TOPIC: (‘cohort stud*’ or ‘longitudinal stud*’ or ‘follow up stud*’ or ‘prospective stud*’ or ‘retrospective stud*’ or ‘controlled clinical trial*’ or ‘Randomized controlled trial*’ or RCT or Random* or blind or case OR 'case control stud*')
10. TOPIC: (hand* OR aseptic* OR intervent* OR program* OR strategy* OR hygiene* OR clean* OR control OR prevention OR screen* OR wash OR protect* OR isolation OR sanitation)
11. TOPIC: ('Long-Term Care' or 'Assisted-Living Facilities' or 'long-term-care facility' or 'Homes for the Aged' or 'Nursing Homes' or 'nursing home' or 'long-term care' or 'retirement home')
12. #7 AND #8 AND #9 AND #10 AND #11

**Supplement 4**. Cochrane search strategy

1. ('hospital acquired' OR nosocomial*):ti,ab,kw
2. ('clostridium difficile' or 'c difficile' or 'c-difficile' or 'c-diff' or 'c diff' or clostrid* Carbape* or 'hospital acquired' or 'Cross infection' or nosocomial* or iatrog*):ti,ab,kw
3. ('Urinary-Tract Infections' OR 'Urinary-Tract Infection' OR 'Blood-Borne Pathogens' OR 'acquired pneumonia' OR pneumonia OR 'associated pneumonia' OR flu OR cold):ti,ab,kw
4. MeSH descriptor: [Clostridium] explode all trees
5. (Clostridium):ti,ab,kw
6. (Clostridium Infections):ti,ab,kw
7. MeSH descriptor: [Clostridium Infections] explode all trees
8. ('COVID 19' OR 'corona virus' OR 'Respiratory syncytial virus' OR 'Respiratory infection' OR 'Respiratory infections'):ti,ab,kw
9. ('Respiratory syncytial virus'):ti,ab,kw
10. ('Haemophilus influenzae' OR 'Respiratory viruses' OR 'Influenza viruses' OR 'Parainfluenza viruses' OR Adenoviruses OR 'Escherichia coli' OR Shigella OR Rotaviruses OR Noroviruses OR Salmonella OR Rhinoviruses OR Chlamydia pneumoniae OR Enterovirus):ti,ab,kw
11. ((vancomycin) AND (resistant OR Enteroc*)):ti,ab,kw
12. (Bacil* AND Gram AND Neg*):ti,ab,kw
13. MeSH descriptor: [Vancomycin-Resistant Enterococci] explode all trees
14. MeSH descriptor: [Methicillin-Resistant Staphylococcus aureus] explode all trees
15. (methicillin):ti,ab,kw AND ('Staphylococcus aureus'):ti,ab,kw
16. MeSH descriptor: [Staphylococcus aureus] explode all trees
17. (vancomycin):ti,ab,kw AND (Enteroc*):ti,ab,kw
18. (Gastrointestinalis OR Gastrointestinal OR gastroenteritis):ti,ab,kw
19. MeSH descriptor: [Tuberculosis, Gastrointestinal] explode all trees
20. MeSH descriptor: [Gastrointestinal Diseases] explode all trees
21. (Tuberculosis):ti,ab,kw
22. MeSH descriptor: [Tuberculosis] explode all trees
23. MeSH descriptor: [Mycobacterium tuberculosis] explode all trees
24. MeSH descriptor: [Tuberculosis, Multidrug-Resistant] explode all trees
25. MeSH descriptor: [Latent Tuberculosis] explode all trees
26. MeSH descriptor: [Extensively Drug-Resistant Tuberculosis] explode all trees
27. (scabies or 'Skin and Connective Tissue Diseases' or 'skin diseases' or 'skin infections' or 'parasitic diseases'):ti,ab,kw
28. MeSH descriptor: [Scabies] explode all trees
29. MeSH descriptor: [Skin and Connective Tissue Diseases] explode all trees
30. MeSH descriptor: [Skin Diseases, Infectious] explode all trees
31. MeSH descriptor: [Skin Diseases] explode all trees
32. MeSH descriptor: [Skin Diseases, Parasitic] explode all trees
33. ('conjunctivitis'):ti,ab,kw
34. MeSH descriptor: [Conjunctivitis] explode all trees
35. MeSH descriptor: [Conjunctivitis, Viral] explode all trees
36. MeSH descriptor: [Conjunctivitis, Bacterial] explode all trees
37. MeSH descriptor: [Conjunctivitis, Allergic] explode all trees
38. MeSH descriptor: [Conjunctivitis, Acute Hemorrhagic] explode all trees
39. MeSH descriptor: [Conjunctivitis, Inclusion] explode all trees
40. MeSH descriptor: [Legionella] explode all trees
41. MeSH descriptor: [Legionella pneumophila] explode all trees
42. MeSH descriptor: [Legionnaires' Disease] explode all trees
43. ('Legionella'):ti,ab,kw
44. #1 OR #2 OR #3 OR #4 OR #5 OR #6 OR #7 OR #8 OR #9 OR #10 OR #11 OR #12 OR #13 OR #14 OR #15 OR #16 OR #17 OR #18 OR #19 OR #20 OR #21 OR #22 OR #23 OR #24 OR #25 OR #26 OR #27 OR #28 OR #29 OR #30 OR #31 OR #32 OR #33 OR #34 OR #35 OR #36 OR #37 OR #38 OR #39 OR #40 OR #41 OR #42 OR #43
45. (price or prices or pricing or expenditure or expenditures or expense or expenses or finan* or fees or charges or Budget or budgets or cost* or econom* analysis or efficienc* or cost effect* or cost utility or cost benefi* or cost consequenc* or cost effic* or cost minimizat*):ti,ab,kw
46. MeSH descriptor: [Costs and Cost Analysis] explode all trees
47. MeSH descriptor: [Cost-Benefit Analysis] explode all trees
48. #45 OR #46 OR #47
49. ('cohort stud*' or 'longitudinal stud*' or 'follow-up stud*' or 'prospective stud*' or longitudinal or prospective or retrospective or 'controlled clinical trial*' or 'Randomized controlled trial*' or RCT or Control* or Random* or blind or case control* or Case* or cohort* or longitudinal*):ti,ab,kw
50. MeSH descriptor: [Cohort Studies] explode all trees
51. MeSH descriptor: [Longitudinal Studies] explode all trees
52. MeSH descriptor: [Follow-Up Studies] explode all trees
53. MeSH descriptor: [Prospective Studies] explode all trees
54. MeSH descriptor: [Retrospective Studies] explode all trees
55. MeSH descriptor: [Controlled Clinical Trial] explode all trees
56. MeSH descriptor: [Randomized Controlled Trial] explode all trees
57. MeSH descriptor: [Case-Control Studies] explode all trees
58. #49 OR #50 OR #51 OR #52 OR #53 OR #54 OR #55 OR #56 OR #57
59. ('Long-Term Care' OR 'Assisted-Living Facilities' OR 'long-term-care facility' OR 'Homes for the Aged' OR 'Nursing Homes' OR 'nursing home' OR 'long-term care' OR retirement home):ti,ab,kw
60. MeSH descriptor: [Residential Facilities] explode all trees
61. #59 OR #60
62. (Hand* or Aseptic* or intervent* or Program* or Strateg* or hygiene* or Clean* or control or prevention or screen* or wash or protect* or isolation or sanitation):ti,ab,kw
63. MeSH descriptor: [Hand Sanitizers] explode all trees
64. MeSH descriptor: [Hygiene] explode all trees
65. MeSH descriptor: [Hand Disinfection] explode all trees
66. MeSH descriptor: [Patient Isolation] explode all trees
67. MeSH descriptor: [Sanitation] explode all trees
68. #62 OR #63 OR #64 OR #65 OR #66 OR #67
69. #44 AND #48 AND #58 AND #61 AND #68

**Supplement 5**. Cinahl search strategy

1. TI ('clostridium difficile' OR 'c difficile' OR 'c-difficile' OR 'c. difficile' OR c diff' OR 'c-diff' OR 'c. diff' OR clostrid* Carbape* OR "hospital acquired" OR "Cross infection" or nosocomial* OR iatrog*) OR AB ('clostridium difficile' OR 'c difficile' OR 'c-difficile' OR 'c. difficile' OR c diff' OR 'c-diff' OR 'c. diff' OR clostrid* OR Carbap* OR "hospital acquired" OR "Cross infection" OR nosocomial* OR iatrog*)
2. TI ('Urinary-Tract Infections' OR 'Urinary-Tract Infection' OR 'Blood-Borne Pathogens' OR 'acquired pneumonia' OR pneumonia OR 'associated pneumonia' OR flu OR cold) OR AB ('Urinary-Tract Infections' OR 'Urinary-Tract Infection' OR 'Blood-Borne Pathogens' OR 'acquired pneumonia' OR pneumonia OR 'acquired pneumonia' OR 'associated pneumonia' OR flu OR cold)
3. (MM "Urinary Tract Infections, Catheter-Related") OR (MM "Urinary Tract Infections+")
4. (MM "Bloodborne Pathogens") OR (MM "Pneumonia, Pneumocystis") OR (MM "Pneumonia, Viral") OR (MM "Pneumonia, Aspiration") OR (MM "Community-Acquired Pneumonia") OR (MM "Pneumonia, Bacterial+") OR (MM "Healthcare-Associated Pneumonia") OR (MM "Pneumonia+")
5. TI (Gastrointestinalis OR Gastrointestinal OR gastroenteritis) OR AB (Gastrointestinalis OR Gastrointestinal OR gastroenteritis)
6. TI ('Haemophilus influenzae' OR 'Respiratory viruses' OR 'Influenza viruses' OR 'Parainfluenza viruses' OR Adenoviruses OR 'Escherichia coli' OR Shigella OR Rotaviruses OR Noroviruses OR Salmonella OR Rhinoviruses OR Chlamydia pneumoniae OR Enterovirus) OR AB ('Haemophilus influenzae' OR 'Respiratory viruses' OR 'Influenza viruses' OR 'Parainfluenza viruses' OR Adenoviruses OR 'Escherichia coli' OR Shigella OR Rotaviruses OR Noroviruses OR Salmonella OR Rhinoviruses OR Chlamydia pneumoniae OR Enterovirus)
7. (MM "Gastroenteritis+") OR (MM "Haemophilus Influenzae") OR (MM "Haemophilus Infections+")
8. (MM "Respiratory Syncytial Viruses") OR (MM "Respiratory Syncytial Virus Infections") OR (MM "SARS Virus") OR (MM "Escherichia Coli") OR (MM "Escherichia Coli Infections") OR (MM "Shigella") OR (MM "Dysentery, Bacillary") OR (MM "Rotaviruses") OR (MM "Rotavirus Infections")
9. (MM "Chlamydophila Pneumoniae") OR (MH "Salmonella Infections") OR (MM "Caliciviridae Infections")
10. (MM "Legionella") OR (MM "Enterovirus Infections+")
11. TI(Legionella) OR AB(Legionella)
12. TI ('COVID 19' OR 'corona virus' OR 'Respiratory syncytial virus' OR 'Respiratory infection' OR 'Respiratory infections') OR AB ('COVID 19' OR 'corona virus' OR 'Respiratory syncytial virus' OR 'Respiratory infection' OR 'Respiratory infections')
13. (MH "Carbapenem-Resistant Enterobacteriaceae")
14. (MH "Clostridium Infections+")
15. (MM "Iatrogenic Disease")
16. (MH "Cross Infection+")
17. (MM "Tuberculosis, Osteoarticular") OR (MM "Tuberculosis, Ocular") OR (MM "Tuberculosis, Gastrointestinal")
18. TI ( (Tuberculosis, Osteoarticular) OR (Tuberculosis, Ocular) OR (Tuberculosis, Gastrointestinal) ) OR AB ( (Tuberculosis, Osteoarticular) OR (Tuberculosis, Ocular) OR (Tuberculosis, Gastrointestinal) )
19. (MM "Scabies") OR (MM "Skin and Connective Tissue Diseases") OR (MM "Skin Diseases") OR (MM "Skin Diseases, Infectious") OR (MM "Skin Diseases, Parasitic") OR (MM "Lice Infestations") OR (MM "Larva Migrans") OR (MM "Onchocerciasis") OR (MM "Parasitic Diseases") OR (MM "Ectoparasitic Infestations") OR (MM "Mite Infestations")
20. TI ( (Scabies) OR (Skin and Connective Tissue Diseases) OR (Skin Diseases) OR (Skin Diseases, Infectious) OR (Skin Diseases, Parasitic) OR (Lice Infestations) OR (Larva Migrans) OR (Onchocerciasis) OR (Parasitic Diseases) OR (Ectoparasitic Infestations) OR (Mite Infestations) ) OR AB ( (Scabies) OR (Skin and Connective Tissue Diseases) OR (Skin Diseases) OR (Skin Diseases, Infectious) OR (Skin Diseases, Parasitic) OR (Lice Infestations) OR (Larva Migrans) OR (Onchocerciasis) OR (Parasitic Diseases) OR (Ectoparasitic Infestations) OR (Mite Infestations) )
21. (MM "Conjunctivitis") OR (MM "Conjunctivitis, Acute Hemorrhagic") OR (MM "Conjunctivitis, Allergic") OR (MM "Conjunctivitis, Bacterial") OR (MM "Conjunctivitis, Inclusion") OR (MM "Conjunctivitis, Viral")
22. TI ((Conjunctivitis) OR (Conjunctivitis, Acute Hemorrhagic) OR (Conjunctivitis, Allergic) OR (Conjunctivitis, Bacterial) OR (Conjunctivitis, Inclusion) OR (Conjunctivitis, Viral)) OR AB ( (Conjunctivitis) OR (Conjunctivitis, Acute Hemorrhagic) OR (Conjunctivitis, Allergic) OR (Conjunctivitis, Bacterial) OR (Conjunctivitis, Inclusion) OR (Conjunctivitis, Viral))
23. #1 OR #2 OR #3 OR #4 OR #5 OR #6 OR #7 OR #8 OR #9 OR #10 OR #11 OR #12 OR #13 OR #14 OR #15 OR #16 OR #17 OR #19 OR #20 OR #21 OR #22
24. (TI Staphylococcus aureus OR AB Staphylococcus aureus) AND (TI methicillin OR AB methicillin)
25. TI ( VRE OR ERV ) OR ( VRE OR ERV )
26. (MH "Methicillin-Resistant Staphylococcus Aureus")
27. (TI Enteroc* OR AB Enteroc*) AND (TI vancomycin OR AB vancomycin )
28. (MH "Vancomycin Resistant Enterococci")
29. (TI Bacil* OR AB Bacil*) AND (TI Gram OR AB Gram) AND (TI Neg* OR AB Neg* )
30. #24 OR #25 OR #26 OR #27 OR #28 OR #29
31. #23 OR #30
32. TI ( Cost* OR econom* OR 'econom* analysis' OR efficienc* OR 'cost effect*' OR 'cost util*' OR 'cost benefit' OR 'cost consequenc*' OR 'cost effic*' ) OR AB ( Cost* OR 'econom* analysis' OR econom* OR efficienc* OR 'cost effect*' OR 'cost util*' OR 'cost benefit' OR 'cost consequenc*' OR 'cost effic*' or 'cost minimizat*')
33. (MH "Economics+")
34. #32 OR #33
35. TI ( controlled clinical trial* OR Randomized controlled trial* OR RCT OR blind OR case control* OR Case* OR cohort* OR longitudinal* ) OR AB ( controlled clinical trial* OR Randomized controlled trial* OR RCT OR blind OR case control* OR Case* OR cohort* OR longitudinal* OR follow up stud* OR prospective stud* OR retrospective stud*)
36. (MH "Randomized Controlled Trials+") OR (MH "Clinical Trials+")
37. (MM "Case Studies") OR (MH "Case Control Studies+") OR (MH "Matched Case Control")
38. (MH "Prospective Studies+")
39. #35 OR #36 OR #37 OR #38
40. TI ( Hand* OR Aseptic* OR intervent* OR Program* OR Strateg* OR hygiene* OR Clean* OR control OR prevention OR screen* OR wash OR protect* OR isolation OR sanitation ) OR AB ( Hand* OR Aseptic* OR intervent* OR Program* OR Strateg* OR hygiene* OR Clean* OR control OR prevention OR screen* OR wash OR protect* OR isolation OR sanitation )
41. (MH "Handwashing+") OR (MM "Infection Control") OR (MM "Hygiene") OR (MH "Patient Isolation+")
42. #40 OR #41
43. TI ('Long-Term Care' OR 'Assisted-Living Facilities' OR 'long-term-care facility' OR 'Homes for the Aged' OR 'Nursing Homes' OR 'nursing home' OR 'long-term care' OR retirement home) OR AB ('Long-Term Care' OR 'Assisted-Living Facilities' OR 'long-term-care facility' OR 'Homes for the Aged' OR 'Nursing Homes' OR 'nursing home' OR 'long-term care')
44. #31 AND #34 AND #39 AND #42 AND #43

**Supplement 6**. EconLIT search strategy

1. AB,TI("clostridium difficile" OR "c difficile" OR "c-difficile" OR "c. difficile" OR "c diff" OR "c-diff" OR "c. diff" OR clostrid* Carbape* OR "hospital acquired" OR "Cross infection" OR nosocomial* OR iatrog*)
2. AB,TI("Urinary-Tract Infections" OR "Urinary-Tract Infection" OR "Blood-Borne Pathogens" OR "acquired pneumonia" OR pneumonia OR "associated pneumonia" OR flu OR cold)
3. AB,TI(Gastrointestinalis OR Gastrointestinal OR gastroenteritis)
4. AB,TI("Haemophilus influenzae" OR "Respiratory viruses" OR "Influenza viruses" OR "Parainfluenza viruses" OR Adenoviruses OR "Escherichia coli" OR Shigella OR Rotaviruses OR Noroviruses OR Salmonella OR Rhinoviruses OR "Chlamydia pneumoniae" OR Enterovirus) OR AB("Haemophilus influenzae" OR "Respiratory viruses")
5. AB,TI("COVID 19" OR "corona virus" OR "Respiratory syncytial virus" OR "Respiratory infection" OR "Respiratory infections")
6. AB,TI("Scabies" or "skin diseases" or "skin diseases parasitic" or "Lice Infestations" or "Larva Migrans" or "Onchocerciasis" or "Parasitic Diseases" or "Ectoparasitic Infestations" or "Mite Infestations" or "Connective Tissue Diseases")
7. AB,TI("Conjunctivitis, Acute Hemorrhagic" or "Conjunctivitis, Allergic" or "Conjunctivitis, Viral" or "Conjunctivitis" or "Conjunctivitis, Bacterial" or "Conjunctivitis, Inclusion")
8. AB,TI("tuberculosis")
9. AB,TI("legionella")
10. #1 OR #2 OR #3 OR #4 OR #5 OR #6 OR #7 OR #8 OR #9
11. AB,TI("Staphylococcus aureus" AND methicillin)
12. AB,TI(VRE OR ERV)
13. AB,TI(Enteroc* AND vancomycin)
14. AB,TI(Bacil* AND Gram AND Neg*)
15. #11 OR #12 OR #13 OR #14
16. #10 OR #15
17. AB,TI(Cost* OR econom* OR "econom* analysis" OR efficienc* OR "cost effect*" OR "cost util*" OR "cost benefit" OR "cost consequenc*" OR "cost effic*")
18. AB,TI("controlled clinical trial*" OR "Randomized controlled trial*" OR RCT OR blind OR "case control*" OR Case* OR cohort* OR longitudinal* OR "follow-up stud*" OR "prospective stud*" OR "retrospective stud*")
19. AB,TI( Hand* OR Aseptic* OR intervent* OR Program* OR Strateg* OR hygiene* OR Clean* OR control OR prevention OR screen* OR wash OR protect* OR isolation OR sanitation )
20. AB,TI("Long-Term Care" OR "Assisted-Living Facilities" OR "long-term-care facility" OR "Homes for the Aged" OR "Nursing Homes" OR "nursing home" OR "long-term care" OR "retirement home")
21. #16 AND #17 AND #18 AND #19 AND #20

**Supplement 7**. SCOPUS search strategy

1. ( econ* OR cost* OR price OR prices OR pricing OR expenditure OR expenditures OR expense OR expenses OR finan* OR fees OR charges OR budget OR budgets ) AND ( nosocomial OR hai OR 'healthcare-acquired AND infection' OR covid OR infection* ) AND ( hygiene* OR screen* OR isolation OR sanitation ) AND ( long-term OR 'nursing AND home' OR retirement ) AND ( 'cohort AND stud*' OR 'longitudinal AND stud*' OR 'follow-up AND stud*' OR 'prospective AND stud*' OR 'retrospective AND stud*' OR 'controlled AND clinical AND trial*' OR 'randomized AND controlled AND trial*' OR rct OR random* OR blind OR case* ) AND ( LIMIT-TO ( PUBYEAR , 2021 ) OR LIMIT-TO ( PUBYEAR , 2020 ) OR LIMIT-TO ( PUBYEAR , 2019 ) OR LIMIT-TO ( PUBYEAR , 2018 ) OR LIMIT-TO ( PUBYEAR , 2017 ) OR LIMIT-TO ( PUBYEAR , 2016 ) OR LIMIT-TO ( PUBYEAR , 2015 ) OR LIMIT-TO ( PUBYEAR , 2014 ) OR LIMIT-TO ( PUBYEAR , 2013 ) OR LIMIT-TO ( PUBYEAR , 2012 ) OR LIMIT-TO ( PUBYEAR , 2011 ) OR LIMIT-TO ( PUBYEAR , 2010 ) OR LIMIT-TO ( PUBYEAR , 2009 ) OR LIMIT-TO ( PUBYEAR , 2008 ) OR LIMIT-TO ( PUBYEAR , 2007 ) OR LIMIT-TO ( PUBYEAR , 2006 ) OR LIMIT-TO ( PUBYEAR , 2005 ) OR LIMIT-TO ( PUBYEAR , 2004 ) OR LIMIT-TO ( PUBYEAR , 2003 ) OR LIMIT-TO ( PUBYEAR , 2002 ) OR LIMIT-TO ( PUBYEAR , 2001 ) OR LIMIT-TO ( PUBYEAR , 2000 ) )

**Supplement 8**. List of excluded studies at full-text screening

| Study | Reason of exclusion |
| --- | --- |
| Armstrong-Evans M, Litt M, McArthur MA, et al. Control of transmission of vancomycin-resistant Enterococcus faecium in a long-term–care facility. Infection Control & Hospital Epidemiology 1999; 20: 312–7. | Wrong intervention |
| Shorr AF, Zilberberg MD, Wang L, Baser O, Yu H. Mortality and costs in Clostridium difficile infection among the elderly in the United States. infection control & hospital epidemiology 2016; 37: 1331–6. | Wrong setting |
| Piednoir E, Borderan G, Borgey F, et al. Direct costs associated with a hospital-acquired outbreak of rotaviral gastroenteritis infection in a long term care institution. Journal of Hospital Infection 2010; 75: 295–8. | Wrong outcome |
| Oxlade O, Vaca J, Romero E, et al. The long-term health and economic benefits of DOTS implementation in Ecuador. Canadian journal of public health 2006; 97: 14–9. | Wrong intervention |
| Navas E, Torner N, Broner S, et al. Economic costs of outbreaks of acute viral gastroenteritis due to norovirus in Catalonia (Spain), 2010–2011. BMC Public Health 2015; 15: 1–8. | Wrong intervention |
| Fukuda H, Yano T, Shimono N. Inpatient expenditures attributable to hospital-onset Clostridium difficile infection: a nationwide case–control study in Japan. Pharmacoeconomics 2018; 36: 1367–76. | Wrong setting |
| Capitano B, Leshem OA, Nightingale CH, Nicolau DP. Cost effect of managing methicillin‐resistant Staphylococcus aureus in a long‐term care facility. Journal of the American Geriatrics Society 2003; 51: 10–6. | Wrong outcome |
| Brakovich B, Bonham E, VanBrackle L. War on the Spore: C lostridium difficile Disease Among Patients in a Long‐Term Acute Care Hospital. Journal for Healthcare Quality 2013; 35: 15–21. | Wrong outcome |
| Boockvar KS, Gruber‐Baldini AL, Stuart B, Zimmerman S, Magaziner J. Medicare expenditures for nursing home residents triaged to nursing home or hospital for acute infection. Journal of the American Geriatrics Society 2008; 56: 1206–12. | Wrong intervention |
| Musa BM, John D, Habib AG, Kuznik A. Cost‐optimization in the treatment of multidrug resistant tuberculosis in Nigeria. Tropical Medicine & International Health 2016; 21: 176–82. | Wrong setting |
| Gordon L, Edwards H, Courtney M, Finlayson K, Shuter P, Lindsay E. A cost-effectiveness analysis of two community models of care for patients with venous leg ulcers. Journal of wound care 2006; 15: 348–53. | Wrong setting |
| Bosco E, van Aalst R, McConeghy K, et al. PIN37 Influenza and RSV-Attributable Cardiorespiratory Hospitalizations in US Long-Term Care Facilities. Value in Health 2021; 24: S112. | Wrong design |
| van Rijt AM, Dik J-WH, Lokate M, Postma MJ, Friedrich AW. Cost analysis of outbreaks with Methicillin-resistant Staphylococcus aureus (MRSA) in Dutch long-term care facilities (LTCF). Plos one 2018; 13: e0208092. | Wrong outcome |
| Roghmann M-C, Lydecker A, Mody L, Mullins CD, Onukwugha E. Strategies to prevent MRSA transmission in community-based nursing homes: a cost analysis. infection control & hospital epidemiology 2016; 37: 962–6. | Wrong outcome |
| Hurtado AV, Nguyen HT, Schenkel V, et al. The economic cost of implementing antigen-based rapid diagnostic tests for COVID-19 screening in high-risk transmission settings: evidence from Germany. Health Economics Review 2022; 12: 1–10. | Wrong outcome |
| de Beer G, Miller MA, Tremblay L, Monette J. An outbreak of scabies in a long-term care facility: the role of misdiagnosis and the costs associated with control. Infection Control & Hospital Epidemiology 2006; 27: 517–8. | Wrong intervention |
| Capitano B, Nicolau DP. Evolving epidemiology and cost of resistance to antimicrobial agents in long-term care facilities. Journal of the American Medical Directors Association 2003; 4: S90–9. | Wrong design |
| Hutton D, Kolli A, Lynem R, Saint S, Krein K, Mody L. An economic evaluation of the targeted infection prevention (TIP) program to reduce multi-drug resistant organisms (MDROS) & infections in high risk nursing home residents. In: An economic evaluation of the targeted infection prevention (TIP) program to reduce multi-drug resistant organisms (MDROS) & infections in high risk nursing home residents. Long Beach, CA United States., 2016. DOI:10.1111/jgs.14231. | Wrong design |
| Plummer E, Wempe WF. Nursing Home COVID Relief Under QIP’s Performance-Based Formula: Does Performance Actually Matter, and Should It? Med Care Res Rev 2022; 79: 851–60. | Wrong intervention |

**Supplement 9**. Summary of the included studies using the Consolidated Health Economic Evaluation Reporting Standards (CHEERS) checklist

| Authors | Campbell et al. (33) | Church et al. (28) | Hutton et al. (31) | Lee et al. (34) | Li et al. (32) | Marchand et al. (27) | Salmerón et al. (35) | Sansone & Bravo (36) | Trick et al. (29) | Verma et al. (30) |
| --- | --- | --- | --- | --- | --- | --- | --- | --- | --- | --- |
| Year of publication | 2020 | 2002 | 2018 | 2021 | 2018 | 1999 | 2022 | 2023 | 2004 | 2013 |
| Country | Canada | Canada | United States of America | United States of America | Hong Kong | Canada | Spain | USA | United States of America | Canada |
| Title | Active testing of groups at increased risk of acquiring SARS-CoV-2 in Canada: costs and human resource needs | Clinical and Economic Evaluation of Rapid Influenza A Virus Testing in Nursing Homes in Calgary, Canada | Economic Evaluation of a Catheter-Associated Urinary Tract Infection Prevention Program in Nursing Homes | How to Choose Target Facilities in a Region to Implement Carbapenem-resistant Enterobacteriaceae Control Measures | Screening for latent and active tuberculosis infection in the elderly at admission to residential care homes: A cost-effectiveness analysis in an intermediate disease burden area | Cost-effectiveness of screening compared to case-finding approaches to tuberculosis in long-term care facilities for the elderly | Efficiency of Diagnostic Test for SARS-CoV-2 in a Nursing Home | Novel care bundle of established basic and practical approaches greatly reduces urinary tract infections in nursing facility residents without indwelling catheters | Comparison of Routine Glove Use and Contact-Isolation Precautions to Prevent Transmission of Multidrug-Resistant Bacteria in a Long-Term Care Facility | Tuberculosis screening for long-term care: a cost-effectiveness analysis |
| Background and objectives | To estimate costs, human resources and laboratory capacity required for active testing strategies to detect SARS-CoV-2 using RT-PCR in groups at increased risk of infection in Canada. | To evaluate the use of a rapid viral diagnostic service for influenza A in conjunction with the previously outlined regional influenza control measures to determine clinical and resource outcomes for the diagnostic service. | To assess the economic effect and cost effectiveness of a targeted catheter-associated urinary tract infection prevention intervention in the nursing home setting. | To choose in which facilities to implement interventions to provide the highest yield. | In order to bridge the policy and research gap, we conducted a cost-effectiveness study to examine whether and under what circumstance screening strategies are cost-effective compared with no screening strategy for the elderly at admission to RCHEs (residential care homes for the elderly) in Hong Kong. | To determine if the more interventionist approach of screening with the tuberculin test and chemoprophylaxis for high-risk positive reactors to control tuberculosis in long-term care facilities is cost-effective when compared to the case-finding and treatment approach. | To determine the efficiency of the strategy of serialization of  positive serologies for coronavirus on a quarterly basis in order to avoid performing unnecessary  AIDT, sick leave and quarantines. Secondary objective: To evaluate, in employees, if  the COVID-19 infection (PCR or positive IgG serology) is related to age, sex or job position. | The purpose of this QAPI (Quality Assurance and performance improvement) initiative was to prevent UTIs among NF  residents without indwelling catheters following the implementation  of a newly developed care bundle of basic and practical clinical  approaches. | To compare routine glove use by healthcare workers for all residents, without use of contact-isolation precautions, with contact-isolation precautions for the care of residents who had vancomycin-resistant enterococci or methicillin-resistant Staphylococcus aureus isolated from a clinical culture. | To examine the potential yield of admission TB screening for long-term care based on a simulation, and to compare the cost-effectiveness of different strategies from the perspective of the health care system. |
| Population | 3,492,250 (Status quo: 1,169,028 people sampled  Strategy 1 (systematically trace and test contacts, in addition to status quo): 1,325,872 people sampled  Strategy 3 (community health care workers and employees and residents of LTCF): 997,350 people sampled) | 1705 nursing homes residents | 418 nursing home residents with indwelling urinary catheters |  | A hypothetical cohort of the 65-year-old elderly population at admission to residential care |  | Sample of 107 residents and 261 employees - Employers or  institutionalized subjects who are working or residing in the San Vicente de Paúl nursing  home (Albacete, Spain) | 262 NF residents: the initiative was limited to UTIs residents without indwelling  catheters whose diagnosis was confirmed by the presence of criteria  as defined in McGeer’s definitions for infection surveillance | 156 (82 control and 76 intervention) |  |
| Setting | Canadian long-term care facilities | 12 nursing homes | 12 Community-based nursing homes | 462 Chicago metropolitan–area healthcare facilities serving adult inpatients | Residential care homes | long-term care facilities for the elderly | San Vicente de Paúl nursing  home | Gouverneur Health a large  public skilled NF in New York City | Skilled-care unit of a 667-bed acute- and long-term care facility | Long term care facilities |
| Study perspective | Health system perspective |  | Health care system perspective | Hospital perspective, Third-Party payer perspective, Societal perspective | Health service provider perspective | Health-care system perspective (Societal perspective because in Canada) |  |  |  | Health care system perspective |
| Intervention | Active testing of groups at increased risk of acquiring Covid-19 (community health care workers and people at long-term care facilities) | Rapid influenza A virus infection diagnostic service | Targeted infection prevention multimodal intervention program for catheter associated urinary tract infections | Two interventions were modeled: an extensively drug-resistant organism registry plus a Carbapenem-resistant Enterobacteriaceae prevention bundle | 3 screening strategies:  tuberculosis Xpert screening, tuberculosis chest X-ray screening and, latent tuberculosis infection and tuberculosis interferon-gamma release assays and chest X-ray screening | Screening with the tuberculin test plus chemoprophylaxis for those at high risk for tuberculosis | COVID-19 screening strategy for second wave: serialization of positive serologies for coronavirus on a quarterly basis in order to avoid performing unnecessary AIDT (PCR or rapid test of antigens), sick leave, and quarantines | Care bundle consisting of 5 components: (1) close monitoring of staff’s  hand hygiene compliance when handling residents; (2) routine  checking of residents’ hydration status; (3) effective residents’ incontinence  and perineal care; (4) in-house UTI treatment and monitor of  antibiotic use; and (5) daily updates regarding the bundle implementation  progress at morning medical staff huddles | Routine glove-use | Three screening strategies for tuberculosis on entry to long term care facilities |
| Comparators | Status quo (=current strategy) defined based on the testing performed between July 8 and 17, 2020, which includes testing of symptomatic people and limited testing of asymptomatic people (e.g., some individuals with exposure or at high risk of exposure). | 6 control nursing homes and 6 experimental nursing homes | 6 control nursing homes and 6 intervention nursing homes | No intervention scenario | No screening strategy | Intervention screening strategy and current standard of care (case-finding and treatment approach) |  | Baseline status | Contact-Isolation Precautions Section | No screening |
| Study design | Cross-sectional study | Randomized clinical trial | Retrospective analysis of randomized clinical trial | Simulation and modelling | A simplified decision analytic process based on Markov model | Modelling | Retrospective observational study | This Quality assurance and performance improvement initiative consisted of 3 stages: a baseline, an intervention and a follow-up  (Crossover design) | Random allocation of two similar sections of the skilled-care unit to one of the infection-control strategies | Modelling |
| Time horizon | July 8^th^, 2020 – July 17^th^, 2020 | 1998-1999 influenza season | 1 year | 50 trials simulated over 3 years | 20 years | 15 years | June 2020 to 18, December 2020 | 48-month period between April 1, 2018 and March 31, 2022 | 1 year | 4 years |
| Discount rate | Discounting done, but the rate is not specified. |  | Discounting done, but the rate is not specified. |  | The costs and effectiveness outcomes were discounted at an annual rate of 5% and adjusted by half-cycle correction. | 5% |  |  |  | 3% |
| Health outcomes | Number of people sampled | For influenza A + residents: illness duration, antibiotic prescription rates (number of times the drug was prescribed, the doses prescribed & duration of the treatment), length of hospital stay  Rate of hospitalization (experimental vs control)  Attack rate  Mortality rate | QALYs lost related to CAUTI | Number of CRE infections  Number of CRE-attributable deaths  QALYs lost | LYs and QALYs | Incremental costs per case avoided per life-year and per quality-adjusted life-year; Incremental costs per death avoided per life-year and per quality-adjusted life-year; Annualized measurements of health events and the cost impact of screening an annual number of INH related hepatitis and INH-related deaths, an annual number of tuberculosis cases, and tuberculosis-related deaths, and the annual cost increment per 1000 institutionalized patients) | Number of PCR tests, sick day leaves and quarantines avoided | UTI rates | Acquisition of microbial organisms (MRSA, extended-spectrum β-lactamase (ESBL)-producing Klebsiella pneumoniae (KP), ESBL-producing Escherichia coli (EC), Vancomycin resistant enterococci) measured by positive cultures | Number of cases |
| Measurement of effectiveness | Cost-benefit | Cost-effectiveness (cost-utility) | Cost- effectiveness (cost-utility & cost-benefit) | Cost- effectiveness  (cost-utility & cost-benefit) | Cost-effectiveness (cost-utility & cost-benefit) | Cost- effectiveness (cost-utility & cost-benefit) | Cost-saving | Cost-saving | Cost-minimization | Cost-effectiveness (cost-utility) |
| Estimating resources and costs | See Table 2 | - Cost data were obtained from numerous sources  - See Table 2, Table 3 | - Only the costs of the intervention and the costs of health outcomes that the intervention affected were used in our calculations; the costs of standard NH care were not included and were assumed to be equal for the intervention and control NHs (Table 1). Costs and outcomes were calculated for a representative 120-bed NH with 6% of residents having indwelling urinary catheters, using the annual rates observed over the 3-year intervention and applied to all residents with indwelling urinary catheters.  - Intervention costs include the cost of an infection prevention specialist to lead and oversee the intervention program, nursing personnel costs to attend educational activities, nursing time for donning protective equipment, supplies for hand hygiene activities and barrier precautions, and interactive educational materials. We did not include costs of active surveillance for MDROs.  - Disease costs:  CAUTI treatment cost: $1745/infection episode  CAUTI hospitalization cost: $7193/infection episode  hospitalization due to septicemia costs: $19914/infection episode  Infection control annual salary: $55000  Nurse hourly wage: $32  Nurse aide hourly wage: $12  Supplies: $2000  Printing: $361  Table 1 & Table 2 | See supplementary appendix Table 1 | We adopted the Hospital Authority charges as cost parameters for the majority of clinical services. As the new diagnostic  tools, costs per test of Xpert and QFT-GIT were estimated from consumable and manpower costs according to a previous study and quotation of the QIAGEN company in Hong Kong. For the costs of manpower resource in screening, diagnosis, and treatment, average hourly incomes of physician and nurse were used at an advanced level (experience of more than 5 years) and calculated according to the public data from the government and annual census. We estimated the average time of health service per case by interviewing doctors  and nurses through a field investigation in Yuen Chau Kok Chest Clinic in Hong Kong. | See Table 4 | Our analysis assumes the following diagnostic tests cost from the Castilla-La Mancha Health Service: PCR $41.5/unit, RT $5/unit and serology $5.5/unit. The cost of sick leave was calculated according to data from the Provincial Council of Albacete (Spain). | Since a paucity of information exists in the literature with regard  to UTI costs per resident episode in NFs and all of it is limited to catheter-  associated UTIs or UTIs in hospitals,9,17,18 a cost of $ 1,250 per  resident episode was estimated for our facility. This estimate  included diagnostic and follow-up tests, type of antibiotics used and  duration of treatment, extra expenses related to infection prevention  measures as well as medical staff and equipment. | - Paper gown: 0.68$ - Usage estimated by counting number of gowns at the beginning and end of shifts for 3 separate residents on contact-isolation precautions  -100 glove box: 3.45$ - usage estimated based on purchasing records | - Alberta database, literature  - See Table 2 |
| Currency | Canadian dollars | Canadian dollars | United States dollars | United States dollars | United States dollars | Canadian dollars | United States dollars ^a^ | United States dollars ^a^ | United States dollars ^a^ | Canadian dollars |
| Price date | 2020 | 1999 ^a^ | 2015 | 2021 ^a^ | 2018 ^a^ | 1992 | 2020 ^a^ | 2022 ^a^ | 1999 ^a^ | 2010 ^a^ |
| Choice of model |  |  | Monte Carlo analysis for probabilistic sensitivity analysis | Regional Healthcare Ecosystem Analyst – generated agent-based model | Markov Model | Markov Model |  |  |  | Markov Model |
| Assumptions | We assumed 1 complete round of testing would take 28 days for hospitals, community health care workers,  long-term care facilities and essential businesses, and 42 days for the larger population in schools. |  | In the probabilistic sensitivity analysis, the parameters are assumed to be normally distributed, with the base representing the mean, and the minimum and maximum representing a 4–standard deviation spread (roughly 95% of the distribution). | Our initial scenario assumed no intervention, whereas experimental scenarios consisted of implementing both the XDRO  registry and CRE prevention bundle in various groups of target facilities, identified in different ways, up to a set bed limit. These  methods ranged from those considering a single factor or characteristic that may be readily identified (ie, information is currently  or readily available), to those considering one metric that accounts for more complicated factors/characteristics but may  require data analysis, to methods considering a combination of single and/or complicated factors that may or may not require  data analysis. Table 1 describes these methods as well as the characteristics of facilities identified by these selection methods.  We normalized each group of target facilities by facility bed size, so that facilities were chosen up to a budget-limited 1686  beds (only intervening beds counted toward the limit). We did not allow for partial facility selection (all targeted units in a facility  were included); thus, some groups had < 1686 intervening beds. Each method generated 2 groups of target facilities  (Table 1): (1) All regional facilities (N = 462) were eligible for selection, and (2) we applied a geographic constraint to only select  facilities within 13 miles of Rush University Medical Center in Chicago (n = 161). We applied this constraint to explore the  impact of logistical considerations (eg, driving distance based on 1 hour of travel time in traffic) important for intervention planning and decision making. | Within the model, each individual at admission to RCHEs is assigned one of three initial health states: No LTBI, LTBI, and TB. In each one-year Markov cycle, the individual is either stay or transit to another health state until death. The model was designed to capture the essence of TB natural history and impacts of early detection and treatment in strategies by simulating a cohort of aged population through the transition of health states within 20 Markov cycles. | The elderly population in institutions was viewed as a stationary population with a constant and equal number of admissions and deaths each year of the 15-year cohort. The proportion of anergic patients is assumed to be zero. |  |  |  | We assumed that patients who were exposed to TB in long-term care and who subsequently acquired LTBI had a higher reactivation risk, of 1.5% per year.  If patients were prescribed preventive therapy, we assumed a 3% risk of serious side effects requiring hospitalisation. |
| Analytic methods |  |  |  |  |  |  |  | Chi square & ANOVA | Categorical variables were compared using the Fisher exact or chi-square test, and relative risks and 95% confidence intervals were calculated. Continuous variables were compared using the nonparametric Kruskal-Wallis test.  P value |  |
| Study parameters |  |  |  |  |  |  |  |  |  |  |
| Incremental costs and outcomes |  |  | Taking into account uncertainty in all parameter values simultaneously in the probabilistic sensitivity analysis, we conclude that the TIP intervention is 85% likely to be cost saving and 96% likely to be cost effective at a threshold of $200,000/QALY. This cost-effectiveness analysis showed that the TIP intervention program was expected to save $34,000 per year and improve health outcomes by 0·2 QALYs. | Dominant: less costly and more effective compared to no intervention | ICER (US$/LYs):  TB screening (Xpert): $9,076  TB screening (CXR): $13,257  LTBI/TB screening: $32,150  - ICER (US$/QALYs):  TB screening (Xpert): $6,094  TB screening (CXR): $8,935  LTBI/TB screening: $19,712 | See Table 5 |  | An overall saving of $33,907 per quarter  (P < ·01; ANOVA test) |  | -See Table 3  Incremental costs:  LTBI screening: 78,405$  Active TB screening: 531,233$  Incremental outcomes (cases):  LTBI screening: 0·71  Active TB screening: 0·79 |
| Characterizing uncertainty |  |  |  |  |  |  |  |  |  |  |
| Characterizing heterogeneity |  |  |  |  |  |  |  |  |  |  |
| Sensitivity analysis | 1-way sensitivity analysis | Done (results not presented) | Univariate sensitivity analysis on all variables in the model and a probabilistic sensitivity analysis using Monte Carlo simulation |  | Monte Carlo | Univariate sensitivity analysis & multivariate sensitivity analysis using Markov model |  |  |  | One-way sensitivity analysis was performed for all variables assumed. The ranges used were based on 95% confi dence intervals (95%CIs) for data-derived probabilities and reported or reasonable ranges for literature-based values (Table 1). Cost estimates were also subjected to one-way sensitivity analysis (data not shown). |
| Study findings | Active testing strategies can identify a high proportion of people with SARS-CoV-2 infection and minimal or no symptoms.  Our analysis shows that actively testing populations at increased risk of acquiring SARS-CoV-2 in Canada can be feasible. Systematic tracing and testing of 16 contacts per person given a new diagnosis of SARS-CoV-2 infection marginally increases testing costs and could be accomplished with current laboratory capacity. | Our study shows that the new laboratory service provided for the experimental nursing homes significantly diminished the overall duration of outbreaks of influenza A virus infection in nursing homes in the region. | This cost-effectiveness analysis showed that the TIP intervention program was expected to save $34,000 per year and improve health outcomes by 0·2 QALYs. | Targeting LTACHs decreased the prevalence of carriage by a relative 17% and 22% regionwide and within Cook County, respectively, regardless of constraints. | Although no screening offered the greatest cost-saving, LTBI/TB screening was the most effective strategy with highest LYs and QALYs gained and more likely to be cost-effective under the WTP threshold of US$50,000 per QALY gained. | Screening improves the health of the average patient in both baseline and sensitivity analysis. | The serological serialization of coronavirus on a quarterly basis, in residents and employees of our nursing home, has proven to be efficient in avoiding unnecessary expenditure of AIDT during a coronavirus outbreak, as well as avoiding quarantines and sick leave of participants with positive IgG | Thus, the implementation of this novel bundle of care was  successful in (1) achieving a strong decline in UTIs among NF residents without indwelling catheters which met the initiative goal; (2) maintaining UTIs lower than the national rate for more than 2 years which validated the efficacy and sustainability of the bundle; (3) avoiding hospitalizations through in-house treatment of residents; and (4) reducing antibiotic use. The sharp decline in UTIs also generated a net saving of $33,907 per quarter by reducing costs of diagnostic and follow-up tests, use of antibiotics, and extra care expenses related to medical equipment and staff | There was a similar frequency of transmission of antimicrobial-resistant bacteria in the two study sections; there was evidence for resident-to-resident *K. pneumoniae* transmission in the isolation-precautions section. Routine glove use for healthcare workers, which decreases resident social isolation and healthcare facility costs, may be preferable in many long-term care facilities. | Our study found that screening was costly, with large numbers needed to screen to prevent a case. Our analysis indicated that TST screening is more cost-effective than CXR screening for prevalent disease.  Screening all entrants to long-term care for TB may not be cost-effective in a low-burden setting. |
| Journal title | Canadian medical association journal | Clinical Infectious Diseases | The American Geriatrics Society | Clinical Infectious Diseases | PLOS One | International Journal of Epidemiology | Geriatrics | American Journal of Infection Control | American Geriatrics Society | The International Journal of Tuberculosis and Lung Disease |

**Supplement 10.** Quality assessment of studies using SIGN guidelines

| SIGN Criteria | Campbell et al. (33) | | Church et al. (28) | | Hutton et al. (31) | | Lee et al. (34) | | Li et al. (32) | | Marchand et al. (27) | | Salmerón et al. (35) | | Sansone & Bravo (36) | | Trick et al. (29) | | Verma et al. (30) | |
| --- | --- | --- | --- | --- | --- | --- | --- | --- | --- | --- | --- | --- | --- | --- | --- | --- | --- | --- | --- | --- |
| 1.     Is the paper an economic study (i.e. assessing the cost effectiveness of something), or is it just a study of costs? REJECT IF THE LATTER IS TRUE. | Yes | | Yes | | Yes | | Yes | | Yes | | Yes | | Yes | | Yes | | Yes | | Yes | |
| 2.     Is the paper relevant to the key question? Analyse using PICO (Patient or Population Intervention Comparison Outcome). IF NO REJECT (give reason below). IF YES complete the checklist. | Yes | | Yes | | Yes | | Yes | | Yes | | Yes | | Yes | | Yes | | Yes | | Yes | |
| Section 1: Internal validity |  | |  | |  | | | | | | | | | | | | | | | |
| 1.1 The study addresses an appropriate and clearly focused question | Can't say | | Can't say | | Yes | | Yes | | Yes | | Yes | | Can't say | | Yes | | Can't say | | Yes | |
| 1.2 The economic importance of the question is clear | Yes | | Yes | | Yes | | Yes | | Yes | | Yes | | Yes | | Can't say | | No | | Yes | |
| 1.3 The choice of study design is justified | No | | No | | No | | No | | No | | Yes | | No | | Can't say | | No | | Yes | |
| 1.4 All costs that are relevant from the viewpoint of the study are included and are measured and valued appropriately | Yes | | Yes | | Yes | | Yes | | Yes | | Yes | | Yes | | Yes | | Yes | | Yes | |
| 1.5 The outcome measures used to answer the study question are relevant to that purpose and are measured and valued appropriately | Yes | | Yes | | Yes | | Yes | | Yes | | Yes | | Yes | | Yes | | Yes | | Yes | |
| 1.6 If discounting of future costs and outcomes is necessary, it been performed correctly | Can't say | | No | | Can't say | | No | | Yes | | Yes | | No | | No | | No | | Yes | |
| 1.7 Assumptions are made explicit and a sensitivity analysis performed | Yes | | Can't say | | Yes | | Can't say | | Yes | | Yes | | No | | No | | Yes | | Yes | |
| 1.8 The decision rule is made explicit and comparisons are made on the basis of incremental costs and outcomes. | Yes | | Yes | | Yes | | Yes | | Yes | | Yes | | No | | No | | No | | Yes | |
| 1.9 The results provide information of relevance to policy makers | Yes | | Yes | | Yes | | Yes | | Yes | | Yes | | Yes | | Yes | | Yes | | Yes | |
| Section 2: Overall assessment of the study |  | |  | |  | | | | | | | | | | | | | | | |
| Yes | 8 | 72·73% | 7 | 63·64% | 9 | 81·82% | 8 | 72·73% | 10 | 90·91% | 11 | 100% | 6 | 54·55% | 6 | 54·55% | 6 | 54·55% | 11 | 100% |
| Can't say | 2 | 18·18% | 2 | 18·18% | 1 | 9·09% | 1 | 9·09% | 0 | 0% | 0 | 0% | 1 | 9·09% | 2 | 18·18% | 1 | 9·09% | 0 | 0% |
| No | 1 | 9·09% | 2 | 18·18% | 1 | 9·09% | 2 | 18·18% | 1 | 9·09% | 0 | 0% | 4 | 36·36% | 3 | 27·27% | 4 | 36·36% | 0 | 0% |

**Supplement 11.** Quality assessment of studies using Drummond guidelines

| Quality of studies | Campbell et al. (33) | | Church et al. (28) | | Hutton et al. (31) | | Lee et al. (34) | | Li et al. (32) | | Marchand et al. (27) | | Salmerón et al. (35) | | Sansone & Bravo (36) | | Trick et al. (29) | | Verma et al. (30) | |
| --- | --- | --- | --- | --- | --- | --- | --- | --- | --- | --- | --- | --- | --- | --- | --- | --- | --- | --- | --- | --- |
| 1- Clarity of the question being asked | High | | Moderate | | High | | High | | High | | Moderate | | Moderate | | Moderate | | Moderate | | High | |
| 2- Comprehensive description of the competing alternatives | High | | High | | High | | High | | High | | High | | High | | High | | High | | High | |
| 3- How the program’s effectiveness was assessed | High | | High | | High | | High | | High | | High | | High | | High | | High | | High | |
| 4- Identification of costs and consequences of each alternative being compared | High | | High | | High | | High | | High | | High | | High | | High | | High | | High | |
| 5- Accurate measurement of costs and consequences using appropriate physical units | High | | Moderate | | High | | High | | High | | High | | High | | High | | High | | High | |
| 6- Credibility of the assessment of costs and consequences | High | | Moderate | | High | | High | | High | | High | | High | | High | | High | | High | |
| 7- Costs adjusted based on timing: discounting | Moderate | | Low | | Moderate | | Low | | Moderate | | High | | Low | | Low | | Low | | High | |
| 8- Differential analysis of costs and consequences of competing alternatives | High | | High | | High | | Low | | Low | | High | | Low | | Low | | Low | | High | |
| 9- Allowance made for uncertainty in estimates of costs and consequences: Sensibility analysis | High | | Low | | Moderate | | Low | | High | | Moderate | | Low | | Low | | High | | High | |
| 10- Clarity of the presentation and discussion of the results: comparison of results against those of other studies and in other jurisdictions | High | | High | | High | | Moderate | | High | | High | | Moderate | | High | | High | | High | |
| Scores assigned to the quality of studies analyzed (/10) |  | |  | |  | | | | | | | | | | | | | | | |
| High | 9 | 90% | 5 | 50% | 8 | 80% | 6 | 60% | 8 | 80% | 8 | 80% | 5 | 50% | 6 | 60% | 7 | 70% | 10 | 100% |
| Moderate | 1 | 10% | 3 | 30% | 2 | 20% | 1 | 10% | 1 | 10% | 2 | 20% | 2 | 20% | 1 | 10% | 1 | 10% | 0 | 0% |
| Low | 0 | 0% | 2 | 20% | 0 | 0% | 3 | 30% | 1 | 10% | 0 | 0% | 3 | 30% | 3 | 30% | 2 | 20% | 0 | 0% |

**Supplement 12.** Quality assessment of studies using Cochrane guidelines

| Cochrane criteria | Campbell et al. (33) | Church et al. (28) | Hutton et al. (31) | Lee et al. (34) | Li et al. (32) | Marchand et al. (27) | Salmerón et al. (35) | Sansone & Bravo (36) | Trick et al. (29) | Verma et al. (30) |
| --- | --- | --- | --- | --- | --- | --- | --- | --- | --- | --- |
| 1. Is the study population clearly described? | High | High | High | Low | High | Low | High | High | High | Low |
| 2. Are competing alternatives clearly described? | High | High | High | High | High | Moderate | High | Moderate | High | High |
| 3. Is a well-defined research question posed in answerable form? | Moderate | Moderate | High | High | High | Moderate | Moderate | Moderate | Moderate | High |
| 4. Is the economic study design appropriate to the stated objective? | High | High | High | High | High | High | Moderate | High | High | High |
| 5. Is the chosen time horizon appropriate to include relevant costs and consequences? | Low | Moderate | High | High | High | High | Moderate | Moderate | High | High |
| 6. Is the actual perspective chosen appropriate? | High | Low | High | High | High | High | Low | Low | Low | High |
| 7. Are all important and relevant costs for each alternative identified? | High | High | High | High | High | High | High | High | High | High |
| 8. Are all costs measured appropriately in physical units? | High | High | High | High | High | High | High | High | High | High |
| 9. Are costs valued appropriately? | High | Moderate | High | Moderate | Moderate | High | Moderate | Moderate | Moderate | Moderate |
| 10. Are all important and relevant outcomes for each alternative identified? | High | High | High | High | High | High | High | High | High | High |
| 11. Are all outcomes measured appropriately? | High | Moderate | High | High | High | High | Moderate | High | High | High |
| 12. Are outcomes valued appropriately? | High | Low | High | High | High | High | Moderate | High | High | High |
| 13. Is an incremental analysis of costs and outcomes of alternatives performed? | Moderate | Low | Moderate | Low | Low | High | Low | Low | Low | High |
| 14. Are all future costs and outcomes discounted appropriately? | Moderate | Low | Moderate | Low | High | High | Low | Low | Low | High |
| 15. Are all important variables, whose values are uncertain, appropriately subjected to sensitivity analysis? | High | Low | High | Low | High | High | Low | Low | High | High |
| 16. Do the conclusions follow from the data reported? | High | High | High | High | High | High | High | High | High | High |
| 17. Does the study discuss the generalizability of the results to other settings and patient/ client groups? | Low | High | High | Low | Low | Moderate | Low | High | Low | High |
| 18. Does the article indicate that there is no potential conflict of interest of study researcher(s) and funder(s)? | Low | Moderate | High | Low | High | Low | High | High | Low | High |
| 19. Are ethical and distributional issues discussed appropriately? | High | High | Low | High | High | Low | High | Low | Low | High |

| OVERALL | | | | | | | | | | | | | | | | | | | | |
| --- | --- | --- | --- | --- | --- | --- | --- | --- | --- | --- | --- | --- | --- | --- | --- | --- | --- | --- | --- | --- |
| High | 13 | 68·42% | 9 | 47·37% | 16 | 84·21% | 12 | 63·16% | 16 | 84·21% | 13 | 68·42% | 8 | 42·11% | 10 | 52·63% | 11 | 57·89% | 17 | 89·47% |
| Moderate | 3 | 15·79% | 5 | 26·32% | 2 | 10·53% | 1 | 5·26% | 1 | 5·26% | 3 | 15·79% | 6 | 31·58% | 4 | 21·05% | 2 | 10·53% | 1 | 5·26% |
| Low | 3 | 15·79% | 5 | 26·32% | 1 | 5·26% | 6 | 31·58% | 2 | 10·53% | 3 | 15·79% | 5 | 26·32% | 5 | 26·32% | 6 | 31·58% | 1 | 5·26% |
